# Clinical applications of stem cell-derived exosomes

**DOI:** 10.1038/s41392-023-01704-0

**Published:** 2024-01-12

**Authors:** Fei Tan, Xuran Li, Zhao Wang, Jiaojiao Li, Khawar Shahzad, Jialin Zheng

**Affiliations:** 1 Department of ORL-HNS, Shanghai Fourth People’s Hospital, and School of Medicine, Tongji University, Shanghai, China; 2 Plasma Medicine and Surgical Implants Center, Tongji University, Shanghai, China; 3 The Royal College of Surgeons in Ireland, Dublin, Ireland; 4 The Royal College of Surgeons of England, London, UK; 5 Center for Translational Neurodegeneration and Regenerative Therapy, Tongji Hospital affiliated to Tongji University School of Medicine, Shanghai, China; 6 Shanghai Frontiers Science Center of Nanocatalytic Medicine, Tongji University, Shanghai, China

**Keywords:** Regeneration, Preclinical research, Diseases

## Abstract

Although stem cell-based therapy has demonstrated considerable potential to manage certain diseases more successfully than conventional surgery, it nevertheless comes with inescapable drawbacks that might limit its clinical translation. Compared to stem cells, stem cell-derived exosomes possess numerous advantages, such as non-immunogenicity, non-infusion toxicity, easy access, effortless preservation, and freedom from tumorigenic potential and ethical issues. Exosomes can inherit similar therapeutic effects from their parental cells such as embryonic stem cells and adult stem cells through vertical delivery of their pluripotency or multipotency. After a thorough search and meticulous dissection of relevant literature from the last five years, we present this comprehensive, up-to-date, specialty-specific and disease-oriented review to highlight the surgical application and potential of stem cell-derived exosomes. Exosomes derived from stem cells (e.g., embryonic, induced pluripotent, hematopoietic, mesenchymal, neural, and endothelial stem cells) are capable of treating numerous diseases encountered in orthopedic surgery, neurosurgery, plastic surgery, general surgery, cardiothoracic surgery, urology, head and neck surgery, ophthalmology, and obstetrics and gynecology. The diverse therapeutic effects of stem cells-derived exosomes are a hierarchical translation through tissue-specific responses, and cell-specific molecular signaling pathways. In this review, we highlight stem cell-derived exosomes as a viable and potent alternative to stem cell-based therapy in managing various surgical conditions. We recommend that future research combines wisdoms from surgeons, nanomedicine practitioners, and stem cell researchers in this relevant and intriguing research area.

## Introduction

**Fig. 1:**
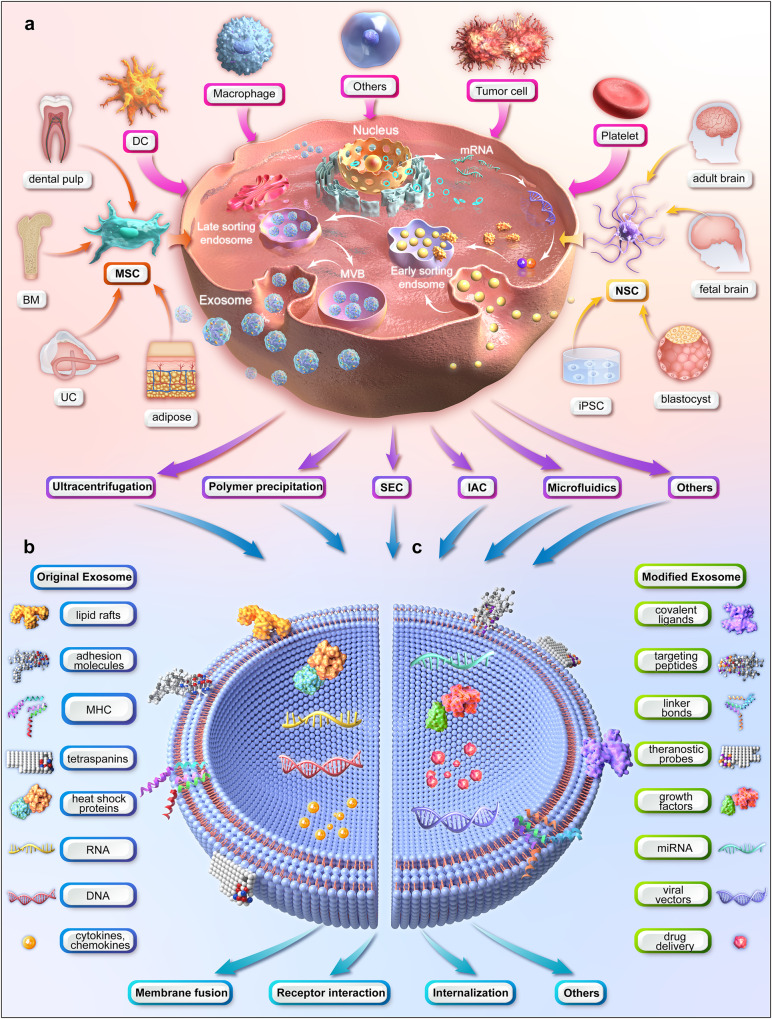
Illustration of the upstream measures of exosome therapy (figure generated using Autodesk 3ds Max 2023). **a** production and purification of exosomes (MSCs and NSCs are used as examples for multipotent stem cells). **b** content of natural exosomes. **c** modification of exosomes. (BM bone marrow, DC dendritic cell, IAC immunoaffinity chromatography, iPSC induced pluripotent stem cell, MHC major histocompatibility complex, miRNA microRNA, MSC mesenchymal stem cell, MVB multivesicular body, NSC neural stem cell, SEC size-exclusion chromatography, UC umbilical cord)

Stem cells are a population of undifferentiated cells with unique abilities to self-renew and recreate functional tissues. They are primarily classified by their differentiation potential, origin and lineage progression. According to their potency, stem cells can be totipotent, pluripotent, multipotent, oligopotent and unipotent.^1^ Stem cells exist both in embryos and adult cells. Embryonic stem cells (ESCs) and induced pluripotent stem cells (iPSCs) are best examples of pluripotent stem cells,^2^ whereas adult multipotent stem cells are exemplified by hematopoietic stem cells (HSCs),^3^ mesenchymal stem cells (MSCs),^4^ neural stem cells (NSCs),^5^ and endothelial stem/progenitor cells (EPCs)^6^ (Fig. 1a). All these subtypes of stem cells have been extensively trialed for the treatment of human diseases.

Stem cell-based therapy, as a modality of regenerative medicine, has generated tremendous attention, as it offers new options for patients suffering from previously incurable diseases. Subsequently, thousands of related clinical trials have been registered, covering a wide spectrum of medical problems, such as musculoskeletal and neurological disorders, immune diseases, hematological dysfunctions, and degenerative conditions.^7^ However, some trials have failed to show any benefit in the clinic. This is likely due to the inevitable limitations of stem cell therapy, such as infusion toxicity, immunogenicity, tumorigenic potentials and ethical issues.^8^ Exosome, secreted by almost all cell types including stem cells (Fig. 1a), has been posited as a safer and more versatile alternative to stem cell therapy.^9^

Exosomes are nanoscale, spherical, and lipid bi-layered single membrane extracellular vesicles, which act as intercellular messengers.^10^ Exosomes have been regarded as miniature versions of their parental cells, partially because exosomes from a certain cell type provide cell-specific or unique sets of biomolecules. In addition, the stem cells have been found to function in a paracrine fashion through their soluble secretome including exosomes.^11^ In other words, stem cell-derived exosomes (SC-Exo) inherit similar therapeutic effects from their parental cell of origin, e.g., anti-inflammation, immunomodulation and tissue regeneration.^12^ Collectively, stem cell-derived exosomes are a potent surrogate for stem cell therapy without exhibiting the disadvantages their cellular counterparts present^13^ (Table 1).

**Table 1:** The comparison between stem cell therapy and stem cell-derived exosome therapy

Treatment modality	Advantages	Limitations
Stem cell therapy	multilineage differentiation potential	short-lived viability and low engraftment after injection
	applicable to the treatment for a wide range of diseases	stringent storage and transport requirements
	extensive accumulation of laboratory and clinical data	tumorigenic potential
	easy to isolate and possible for mass-production	infusion toxicity
	well-developed regulatory guidelines	immunogenicity
		ethical issues
Stem cell-derived exosome therapy	comparable therapeutic effects to stem cells but much smaller	batch-to-batch inconsistency
	more concentrated functional cargos, e.g., cytokines	no standardized protocol for purification and storage
	modifiable at its surface and in its cargos	relatively low yield for large scale manufacturing
	versatile delivery modalities	no industry-standard quality specifications
	stable for long-term storage and transport	insufficient regulatory control
	negligible risk of tumorigenesis and immune response	
	lack of ethical issues	

Prior to clinical applications, exosomes must be prepared and optimized in terms of production, purification, and modification (Sections 2.3 and 2.4). A wide range of medical reviews analyzing these upstream measures of exosome therapy have been published in recent years. Nevertheless, some research avenues remain under-investigated: in particular, systematic investigation dedicated to downstream clinical applications is lacking, especially from a surgical perspective. Tissues that have been damaged, whether by disease or a surgeon’s scalpel, respond by inflammatory and regenerative dynamics,^14^ making surgery a perfect arena for stem cell-derived exosome therapy.^15^ Stem cell-derived exosomes inherit similar therapeutic effects from their parental cell of origin, e.g., tissue regeneration, anti-inflammation and immunomodulation.^12,16–18^

**Fig. 2:**
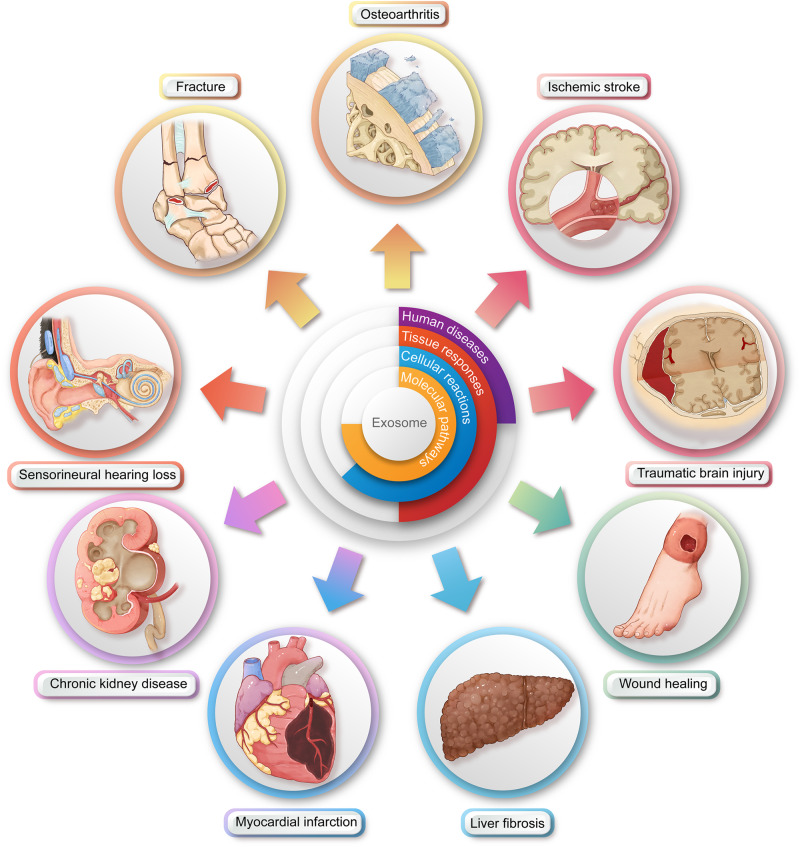
Illustration of the downstream surgical applications of exosome therapy (figure generated using Adobe Photoshop 2023 and Adobe Illustrator 2023). The therapeutic effects of exosomes are a hierarchical translation through disease-specific tissue responses, tissue-specific cellular alterations, and cell-specific molecular signaling pathways

In this work, we will dissect relevant publications from the last five years in order to present a comprehensive, up-to-date, specialty-specific and disease-oriented review (Fig. 2). Our aim is to bridge the gap that currently exists between surgeons, nanomedicine practitioners, and stem cell researchers.

## General background of exosomes and exosome therapy

### Biogenesis, composition, and uptake of exosomes

Exosomes differ from other types of primary extracellular vesicles (e.g., apoptotic bodies and microvesicles) in terms of size, content, and production mechanism.^19^ The most popularly accepted mechanism of exosome formation, i.e., an endosomal route, is as follows (Fig. 1a). The initial endosomes are produced by cell membrane invagination during which the bioactive substances begin to accumulate within the early sorting endosomes. The late sorting endosomes then form multivesicular bodies (MVBs) after a second indentation. Finally, the MVBs fuse with the cell membrane, releasing the carried exosomes to the outside. Non-endosomal route of exosome biogenesis, such as plasma membrane budding, has also been reported.^20^

As the three major exosome databases (i.e., ExoCarta, Vesiclepedia, and EVpedia) summarize, exosomes contain numerous molecules, including proteins, glycoconjugates, lipids, nucleic acids, metabolites, and other bioactive substances (Fig. 1b). The examples of each category and the corresponding functions have been thoroughly reviewed elsewhere.^21,22^ On the one hand, exosomes comprise a complex protein network including external proteins (e.g., tetraspanins, antigen-presenting complexes, and adhesion molecules) and internal proteins (e.g., heat shock proteins, ESCRT machinery, cytokines and chemokines, and membrane transporters).^23^ On the other hand, as the most abundant in human exosomal nucleic acids, microRNA (mRNA) could participate in hematopoiesis, exocytosis, and nerve and vascular regeneration through exosome-mediated cellular communication.^24^

There are various uptake mechanisms once exosomes reach the recipient cell, all of which can be categorized into membrane fusion, receptor interaction, and internalization^21^ (Fig. 1b). Finally, the exosomal cargos are released into the cytoplasm, the process of which depends on the source of the exosome, nature of the cargo, and the metabolic state of the recipient cell.^25^ The entire lifecycle from exosome biogenesis to uptake and intracellular signaling can be tracked using fluorescent, luminescent, and radioactive techniques.^26,27^

### Source and classification of exosomes

Depending on whether exosomes have been artificially modified, they are broadly classified into natural exosomes and engineered exosomes (Section 2.4). Depending on the species of origin, exosomes are divided into animal-derived and plant-derived exosomes. Currently, exosomes are mainly classified according to the type of their parental cells. Almost all types of human cells can produce exosomes. These include, but are not limited to, macrophages, dendritic cells (DCs), platelets, stem cells, and even tumor cells^28^ (Fig. 1a).

For example, macrophage-derived exosomes contribute to disease progression (e.g., diabetes, atherosclerosis and heart failure)^29^ and disease treatment (e.g., cutaneous wound, inflammatory bowel disease, and fungal and viral infection).^30^ However, they seem to play paradoxical roles in suppressing and promoting tumors.^31^ Like DCs, DC-derived exosomes (Dex) could also interact with immune cells (e.g., T cells, B cells, and NK cells) through their surface proteins such as major histocompatibility complexes (MHCs).^32^ Some preclinical and clinical trials have demonstrated the effectiveness and safety of Dex-based immunotherapy for cancers.^33^ Furthermore, tumor-derived exosomes (Tex) not only are involved during tumor proliferation, invasion, metastasis, and immunity but also can be used as biomarkers for cancer diagnosis and treatment.^34^ Lately, Tex has been used as an anti-tumor drug and an antigen presenter for DC vaccination, serving as a promising cell-free cancer immunotherapy.^35^ Finally, the clinical applications of stem cell-derived exosomes will be discussed in detail in the following sections.

Exosomes can be found in all body fluids such as blood, saliva, urine, plasma, tears, semen, amniotic fluid, and even breast milk.^36^ Body fluid-derived exosomes are a highly stable reservoir of disease biomarkers, assisting liquid biopsy in various clinical settings such as cancers, cardiovascular diseases, and perinatal disorders.^37,38^ However, the coexisting contents and availability of each type of body fluid might create challenges to exosome isolation.

### Production, isolation and purification of exosomes

One of the major obstacles preventing exosome-based therapeutics from entering clinical practice is the low yield and efficiency of exosomes. For example, only less than 1 μg exosomal protein could be harvested from 1 ml culture medium in a laboratory setting.^39^ There are various methods of upscaling exosome production, which are categorized into biochemical strategies (e.g., LPS, BMP-2, HIF-1α, and IFN-γ and TNF-α), physical strategies (hypoxia, thermal stress, and starvation), mechanical strategies (shear stress and 3D culturing) and instrumental strategies (hollow-fiber bioreactors and stirred tank bioreactors).^40^

Exosomes are heterogeneous in terms of size, content, surface markers, and source, which makes their isolation difficult. The currently available techniques for exosome isolation and purification are based on their size, surface charge, or immunoaffinity^26^ (Fig. 1a). However, there is no ‘one-fits-all’ approach as these techniques all have advantages and disadvantages.

For example, ultracentrifugation is deemed the gold standard for exosome extraction. Although it requires minimal reagents and expertize, the time consumption, high cost, low efficiency, and lipoprotein co-separation have limited its large-scale use.^41^ Immunoaffinity chromatography is a separation technology based on the specific binding of antibodies and ligands. It is rapid and provides high purity, specificity, and yield. However, the antigen/protein coupling used needs to be expressed on the surface of exosomes.^19^ Size-based isolation techniques mainly refer to ultrafiltration and size-exclusion chromatography, both of which are quick and suitable for large-scale applications. But pore clogging, exosome loss, and low purity are making this method difficult to popularize.^42^ Although no single technique is perfect, combining the above techniques with others (e.g., precipitation-based and microfluidics-based) might be a solution to simultaneously meet multiple requirements for exosome isolation and purification.

### Modification of exosomes

Exosomes can be biochemically modified to broaden, change, or improve their therapeutic effects. The modification of exosomes is classified into internal strategies (e.g., drug loading) and external strategies (e.g., surface modification). On the one hand, exosomes may be an ideal therapeutic carrier to deliver drugs, nucleic acids, and vaccines due to their advantages in stability, non-immunogenicity, and targeting recipient cells.^43^ There are various cargo loading techniques including pre-production loading methods (e.g., transfection, co-incubation, and electroporation) and post-production loading methods (e.g., freeze-thaw cycles, incubation, sonication, extrusion, and hypotonic dialysis) depending on whether they are applied before or after exosome biogenesis^10,26,44–46^ (Fig. 1c). For example, Tian et al. loaded doxorubicin in Dex using electroporation for the treatment of breast cancer.^47^ Kim et al. loaded paclitaxel in RAW 264.7-derived exosomes using incubation and sonication to overcome multidrug resistance in cancer cells.^48^ Ohno et al. loaded antitumor let-7a miRNA in HEK293-derived exosomes using transfection to manage breast cancer.^49^

On the other hand, surface modification of exosomes is exemplified by genetic engineering of exosomal membrane or parental cells, chemical connection of targeting ligands, electrostatic interaction, and magnetic nanoparticle technology.^10^ The main purpose of surface modification is to selectively deliver exosomes to target cells for precise treatment. For example, Alvarez-Erviti et al. modified DCs using genetic engineering to express Lamp2b and RVG peptides, thereby targeting the central nervous system (CNS).^50^ Zhu et al. inserted tumor-targeting peptides, c(RGDyK), into the exosome surface using a chemical reaction to target glioblastoma.^51^ Nakase et al. bound exosomes with a complex formed by pH-sensitive fusion peptide and cationic lipid using electrostatic interaction, thereby achieving enhanced cytosolic delivery.^52^

### Characterization and verification of exosomes

Exosomes need to undergo characterization and verification before therapeutic applications. Current methods used for exosome characterization mainly focus on the size, morphology, and cargo profile of exosomes.^43^ Size-oriented verification includes nanoparticle tracking analysis (NTA), dynamic light scattering (DLS), and tunable resistive pulse sensing (TRPS), whereas morphology-oriented analysis includes scanning electron microscopy (SEM) and transmission electron microscopy (TEM).^19^ In addition, cargo profiling is further subdivided into proteomic, lipidomic, and genomic analyses including western blotting, ELISA, flow cytometry, mass spectroscopy, and PCR.^36^ Since each of the above characterization methods has advantages and disadvantages, it is a universal practice to combine analyses from three different aspects, e.g., a package of TEM, NTA, and western blotting, to identify isolated exosomes.

For example, microscopy-based methods, such as SEM and TEM, can directly visualize the surface topography and internal structure, respectively. However, TEM is not suitable for quick measurement of a large number of samples due to complicated operation and tedious sample preparation.^53^ NTA facilitates fast detection and real-time exosome observation while having a higher resolution than flow cytometry. The main disadvantage of NTA is its difficulty in distinguishing exosomes from contaminated proteins.^54^ As a mature technique, western blotting can qualitatively and quantitatively detect the expression of exosomal protein biomarkers, especially exosomes from cell culture media. However, it is time-consuming and not suitable for the detection of exosomes from biological fluids.^55,56^

### Storage of exosomes

The currently used preservation methods for long-term storage of exosomes mainly include cryopreservation, lyophilization, and spray-drying.^10^ Temperature and antifreeze are the two most important ingredients for cryopreservation. Storage at 4 °C might weaken the biological activity and reduce the protein cargo of exosomes, whereas −80 °C is considered the optimal temperature causing the least impact on exosome morphology and content.^57,58^ Non-permeable disaccharide antifreeze, especially trehalose, represents the best choice as it prevents exosome aggregation and cryodamage.^59^ Heat-sensitive materials, e.g., exosomes and vaccines, treated by lyophilization of freeze-drying can be easily stored and reconstituted by simply adding water. A recent study showed that lyophilization with cryoprotectant could retain the activity of exosomal proteins and RNA for approximately 4 weeks even when stored at room temperature.^60^ Finally, in contrast to freeze-drying, spray-drying is a single-step process, thereby reducing the need for expensive equipment and lengthy multi-step milling. However, core parameters of spray-drying such as exosome feeding rate, atomization pressure, and outlet temperature, can all affect exosome stability and cargo integrity.^61^

## Orthopedic and trauma surgery and SC-Exo therapy

### Fracture

Fractures are the most common traumatic large-organ injuries, and approximately 10% heal improperly.^62^ Fracture healing involves an anabolic tissue-bulking phase and a catabolic tissue-remodeling phase, which are controlled by various factors such as stem cells, innate and adaptive immune functions, and stability.^63^ Biopharmacological treatment for fractures can be given locally (e.g., bone morphogenetic protein, BMP) or systemically (e.g., parathyroid hormone, PTH). As a promising alternative, exosome therapy for fracture healing mostly utilizes bone marrow-derived MSCs as a cellular supplier (Table 2).

**Table 2:** Stem cell-derived exosomes for the treatment of diseases in orthopedic surgery and related specialties

Target disease	Exosome	In vitro model & findings	In vivo model & findings	Refs.
Fracture	BM-MSC-exo	N/A	femoral fracture model, wild type and CD9^-^/^-^ mice model; rescued retardation of fracture healing in CD9^-^/^-^ mice; promoted bone healing in wild-type mice	^65^
	BM-MSC-exo	HUVECs, MC3T3-E1 cells; improved proliferation & migration	rat model of femoral nonunion; enhanced osteogenesis and angiogenesis via BMP-2/Smad1/RUNX2 pathway	^66^
	young BM-MSC-exo	older BM-MSCs; enhanced proliferation & osteogenic differentiation	distraction osteogenesis rat model; accelerated bone regeneration with better mechanical properties in tibias	^67^
	EPC-exo	HUVECs; enhanced proliferation, migration via miR-126	distraction osteogenesis rat model; accelerated bone regeneration with better mechanical properties in tibias with higher vascular density	^68^
	BM-MSC-exo	MC3T3-E1 cells; promoted proliferation & differentiation	mice; exosomal miR-136-5p promoted fracture healing by targeting LRP4 to activate Wnt/β-catenin pathway	^70^
	BM-MSC-exo	MC3T3-E1 cells; accelerated osteogenic differentiation, proliferation, and migration	mice; exosomal miR-25 regulated ubiquitination and degradation of Runx2 by SMURF1 to promote fracture healing	^71^
	BM-MSC-exo	BM-MSCs; high-fat diet inhibited exo secretion & osteogenic markers	obesity-induced fracture mouse model; exosomal lncRNA H19 improved fracture healing via miR-467/HoxA10 axis	^72^
	BM-MSC-exo	HUVECs, BM-MSCs; promoted angiogenesis & osteogenesis via angiopoietin-1/Tie2-NO pathway	CBS-heterozygous mice; exosomal lncRNA-H19 absorbed miR-106 and restored bone formation and mechanical quality	^73^
	DMOG-stimulated BM-MSC-exo	HUVECs; promoted proliferation and tube formation	calvarial defect rat model; improved bone regeneration and neovascularization by activating Akt/mTOR pathway	^74^
	BM-MSC-exo	HUVECs; exosomal miR-29a promoted proliferation, migration, and tube formation by vasohibin-1	mice; miR-29a-loaded exo promoted angiogenesis and osteogenesis by increasing trabecular bone mass	^75^
	UC-MSC-exo	HUVECs; hypoxia enhanced exo production via HIF-1α; improved proliferation & tube formation	femoral fracture mouse model; hypoxic exo promoted fracture healing by transferring miR-126 to a greater extent than normoxic exo	^76^
Osteoporosis	adipose-MSC-exo	MLO-Y4 cells; reduced hypoxia/serum deprivation-induced osteocyte apoptosis and osteocyte-mediated osteoclastogenesis	N/A	^118^
	UC-MSC-exo	BM-MSCs; inhibited apoptosis	HLU-induced disuse osteoporosis rat model; acted via miR-1263/Mob1/Hippo signaling pathway	^119^
	UC-MSC-exo	osteoblasts; promoted cell proliferation and osteogenic differentiation	estrogen-deficient osteoporosis model mice; improved tibial density and reversed osteoporosis; miR-2110 and miR-328-3p are most important osteogenesis regulatory exosomal mRNAs	^120^
Osteoarthritis	IPFP-MSC-exo	chondrocytes; inhibited apoptosis & autophagy, and enhanced matrix synthesis	mice; exosomal miR-100-5p ameliorated OA severity by protecting articular cartilage and ameliorating gait abnormalities via inhibition of mTOR	^79^
	MSC-exo	chondrocytes; promoted proliferation and inhibited apoptosis	mice; exosomal lncRNA-KLF3-AS1 protected chondrocytes via miR-206/GIT1 axis	^80^
	synovial-MSC-exo	primary chondrocytes; miR-320c-enhanced chondrogenesis via ADAM19	N/A	^81^
	chondrogenic MSC-exo	chondrocytes; increased cell proliferation and matrix synthesis via targeting Wnt5a	mice; exosomal miR-92a-3p inhibited cartilage degradation in OA animal model	^82^
	synovial-MSC-exo	human primary chondrocytes; enhanced proliferation & migration via Wnt/YAP signaling	rats; miR-140-5p-oe-exo prevented OA by decreasing joint wear and cartilage matrix loss	^83^
	TGF-β-stimulated MSC-exo	C5.18 cells; exosomal miR-135b increased cell viability by regulating specificity protein-1	rats; promoted cartilage repair by decreasing OARSI score and increasing number of chondrocytes	^84^
	iPSC-exo, MSC-exo	human chondrocytes; stimulated proliferation & migration	collagenase-induced OA mice; iPSC-MSC-exo showed a stronger therapeutic effect on OA than synovial membrane MSC-exo	^85^
	BM-MSC-exo	chondrocytes; decreased inflammatory factors & glutamine metabolic proteins	rats; increased mice’s exercise capacity, improved chondrocyte function and glutamate metabolism, and decreased cartilage damage and inflammation, thereby alleviating OA progression	^86^
	BM-MSC-exo	osteoblasts; promoted cell proliferation and osteogenic differentiation by reducing Elf3	mice; exosomal miR-206 ameliorated inflammation and increased osteocalcin and BMP2 in femoral tissue;	^87^
	MSC-exo	chondrocytes; increased proliferation, matrix synthesis and regenerative immune phenotype	rat osteochondral defect model; ↑ CD163^+^ M2 and ↓ CD86^+^ M1 macrophages, and reduced pro-inflammatory cytokines IL-1β & TNF-α	^88^
	gingival-MSC-exo	CD4^+^ T-cells; inhibited IL-17A and promoted IL-10	collagen-induced arthritis mice model; reduced incidence and bone erosion of arthritis via inhibiting IL-17RA-Act1-TRAF6-NF-κB pathway	^89^
Spinal cord injury	IGF-1 stimulated NSC-exo	PC12 cells; inhibited apoptosis and promoted neural proliferation & regeneration	rats; reduced lesion size and promoted functional recovery, caused by miR-219a-2-3p-dependent inhibition of YY1	^94^
	miR-enclosed NSC-exo	HT22 cells; attenuated neuronal apoptosis by activating autophagy via miR-374-5p/STK-4 axis	mice, subarachnoid injection; enhanced functional recovery	^95^
	MSC-exo	HT-22 & HEK-293 hypoxic cell model; suppressed neuronal ferroptosis	mice; enhanced repair of neurological functions via lncGm36569/miR-5627-5p/FSP1 axis	^96^
	BM-MSC-exo	macrophages; taken up by a subset of M2 macrophages	rats; both MSC intravenous infusion and fractionated MSC-exo promoted M2 macrophage polarization, upregulated TGF-β, and reduced BSCB leakage	^97^
	hypoxic preconditioned BM-MSC-exo	BV2 microglia; hypoxia promoted exo release from MSC; exo uptake by BV2 depended on oxygen status	mice; promoted functional behavioral recovery by shifting microglial M1/M2 polarization; exosomal miR-216a-5p regulated via TLR4/NF-κB/PI3K/Akt pathway	^98^
	EF-MSC-exo	N/A	rats; improved neurological functional recovery and reduced lesion volume by inhibiting NLRP3 inflammasome	^99^
	EPC-exo	macrophages; promoted anti-inflammatory macrophages	mice; exosomal miR-222-3p promoted functional repair via SOC3/JAK2/STAT3 pathway	^100^
	NSC-exo	SCMECs; enriched in VEGF-A and enhanced angiogenic activity	mice; accelerated microvascular regeneration, reduced spinal cord cavity, and improved functional recovery	^101^
	FTY720-loaded NSC-exo	SCMECs; protected barrier function of SCMECs under hypoxic conditions via PTEN/Akt pathway	rats; ameliorated hindlimb function and reduced inflammatory infiltration by downregulating Bax and aquaporin-4 and upregulating claudin-5 and Bcl-2	^102^
	BM-MSC-exo	pericyte; pre-Tx with exo reduced pericyte pyroptosis and increased pericyte survival rate	rat model of T10 SCI; improved neuron survival, nerve fiber extension, BSCB integrity, reduced caspase 1 & IL-1β, and accelerated locomotor functional recovery	^104^
	miR-modified UC-MSC-exo	PC12 cells; reduced negative effects of neurotoxic astrocytes on PC12 cell viability and neurites	rats; miR-146a-5p-modified exo promoted more locomotor function of hindlimbs than unmodified exo by targeting neurotoxic astrocytes	^105^
	BM-MSC-exo-oe-NGF	NSC; promoted differentiation of NSCs into neurons & axonal regeneration	mice; promoted recovery of spinal function & spinal cord regeneration	^106^
	placental-MSC-exo	NSCs; promoted cell proliferation, and increased phosphorylated levels of MEK, ERK and CREB	rats; promoted endogenous neural stem/progenitor cells proliferation, neurogenesis, and improved locomotor activity and bladder dysfunction	^107^
Sciatic nerve injury	adipose-MSC-exo	DRG neurons; increased neurite outgrowth	rats; enhanced axonal regeneration and walking behavior; discovered neural growth factors transcripts in exo	^122^
	adipose-MSC-exo	Schwann cells; promoted proliferation, migration, myelination, & neurotrophic factors	rats; improved axon regeneration & myelination, and restored denervation muscle atrophy	^123^
	LPS-treated BM-MSC-exo	RAW264.7 cells; enhanced M2 macrophage polarization via TSG-6/NF-κB/NLRP3 pathway	rats; accelerated functional recovery, axon regeneration and remyelination	^124^
Muscle & tendon tear	MSC-exo	C2C12 myoblasts, HUVECs; promoted myogenesis and angiogenesis	mouse model of cardiotoxin-induced muscle injury; promoted muscle regeneration; exosomal miR-494 enhanced myogenesis and migration activity	^111^
	adipose-MSC-exo	rabbit primary tenocytes; enhanced proliferation and migration	Achilles tendon repair rabbit model; improved mechanical strength by upregulating decorin and biglycan	^112^
	adipose-MSC-exo	N/A	rat model of massive rotator cuff tear; prevented atrophy, fatty infiltration, inflammation, and vascularization of muscles; elevated myofiber regeneration and biomechanical properties	^114^
	adipose-MSC-exo	N/A	rabbit model of chronic rotator cuff tear; decreased fatty infiltration, promoted tendon-bone healing, and improved biomechanical properties	^115^
	BM-MSC-exo	HUVECs, U937 cells; promoted proliferation & angiogenic tube formation; reduced M1 polarization	rats; increased breaking load and stiffness of rotator cuff after reconstruction in rats, reduced angiogenesis around rotator cuff endpoint, and promoted tendon-bone healing	^116^
Intervertebral disc degeneration	BM-MSC-exo	nucleus pulposus cells; exosomal miR-21 alleviated apoptosis via PTEN/PI3K/Akt pathway	rats; intradiscal injection of exo alleviated nucleus pulposus apoptosis and IVD degeneration based on histology and MRI	^126^
	ESC-exo	nucleus pulposus cells; exosomal miR-302c inhibited pyroptosis	rats; ameliorated damage in IVD degeneration via downregulating NLRP3 inflammasome	^127^
	BM-MSC-exo	nucleus pulposus cells; alleviated compression-induced apoptosis & mitochondrial damage by inhibiting oxidative stress	N/A	^128^

*Akt* protein kinase B, *BM* bone marrow, *BMP* bone morphogenetic protein, *BSCB* blood-spinal cord barrier, *CBS* cystathionine β-synthase, *CREB* cAMP response element binding, *DMOG* dimethyloxaloylglycine, *DRG* dorsal root ganglion, *EF* epidural fat, *Elf* E74-like factor, *EPC* endothelial progenitor cell, *ERK* extracellular signal-regulated kinase, *ESC* embryonic stem cell, *exo* exosome, *FSP* fibroblast-specific protein, *GIT* G-protein coupled receptor kinase interacting protein, *HIF* hypoxia-inducible factor, *HLU* hind limb unloading, *HUVEC* human umbilical vein endothelial cell, *IGF* insulin growth factor, *IL* interleukin, *IPFP* infrapatellar fat pad, *IVD* intervertebral disc, *KLF* Krüppel-like factor, *LPS* lipopolysaccharide, *LRP* lipoprotein receptor related protein, *MEK* mitogen-activated protein kinase, *miR* microRNA, *mTOR* mechanistic target of rapamycin, *NF-κB* nuclear factor-kappa B, *NGF* nerve growth factor, *NLRP* nucleotide-binding domain-like receptor protein, *NP* nucleus pulposus, *NSC* neural stem cell, *oe* overexpressing, *PI3K* phosphoinositide 3-kinase, *PTEN* phosphatase & tensin homolog, *Runx* runt-related transcription factor, *SCMEC* spinal cord microvascular endothelial cell, *SMURF* smad ubiquitination regulatory factor, *TGF* transforming growth factor, *Tie* tyrosine kinase receptor, *TLR* Toll-like receptor, *TNF* tumor necrosis factor, *TSG* TNF stimulated gene, *Tx* treatment, *UC* umbilical cord, *VEGF* vascular endothelial growth factor, *YAP* yes-associated protein, *YY* yin and yang

**Fig. 3:**
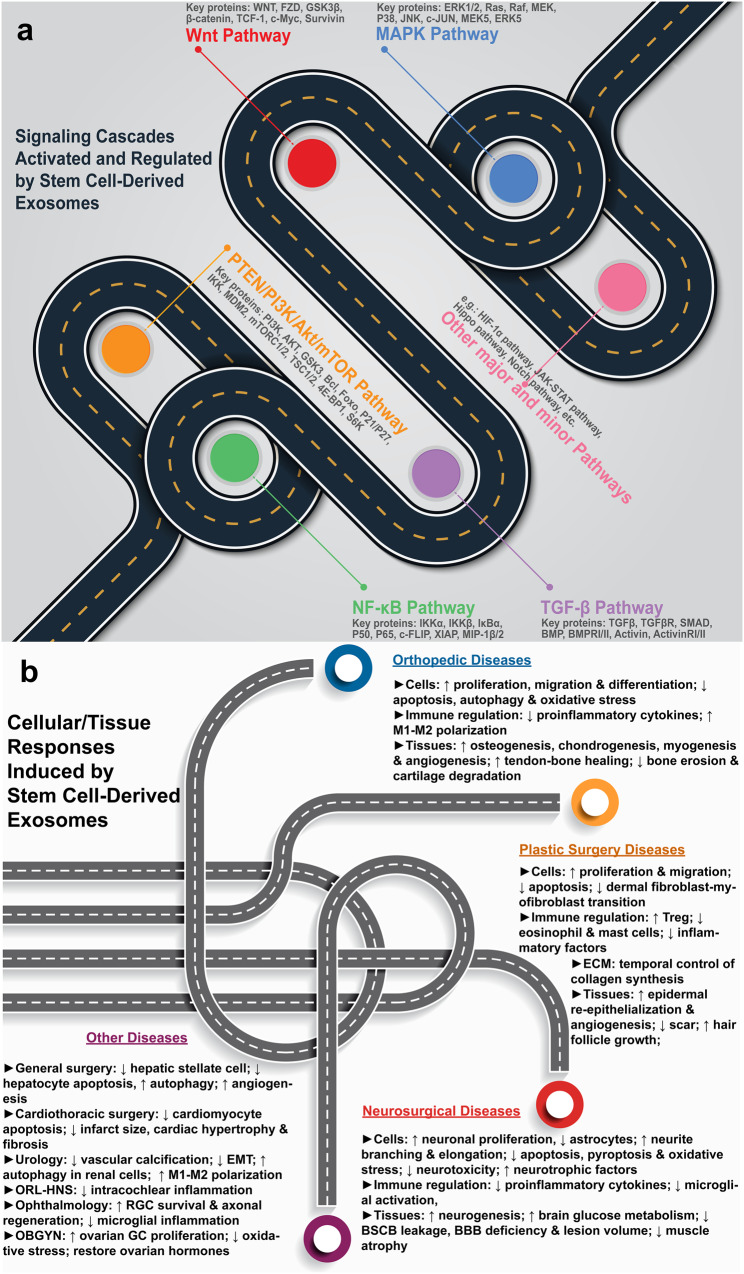
Mechanisms of stem cell-derived exosome therapy (figure generated using Adobe Photoshop 2023 and Adobe Illustrator 2023). **a** activation and regulation of various signaling pathways. **b** disease-specific cellular and tissue responses

The presumed mechanism of how MSC-derived exosomes promote fracture healing is as follows. Firstly, the progression of bone repair needs a variety of cells, e.g., inflammatory cells in the inflammation stage, endothelial and mesenchymal progenitor cells in the fibrovascular stage, osteoblasts and chondrocytes during bone formation, and osteoclasts during callus remodeling.^62^ Secondly, most of these cells can uptake exosomes, especially osteoblasts and vascular endothelial cells,^64^ which are most related to fracture healing. Lastly, upon exosome absorption, the gene expression of the recipient cells is modified, thereby activating various signaling pathways (Fig. 3a), causing various cellular and tissue responses (Fig. 3b) and ultimately leading to improved fracture healing.

Early research has employed various animal models of fracture healing. In a transverse femoral shaft fracture model, exosomes were found to not only promote osteogenesis in wild-type mice, but also rescue retardation of fracture healing in CD9^−^/^−^ mice, a strain known to have a lower bone union rate.^65^ In a femoral nonunion model, exosomes enhanced fracture healing by promoting osteogenesis and angiogenesis possibly via the BMP-2/Smad1/RUNX2 pathway.^66^ In a tibial distraction osteogenesis model, exosomes secreted by young MSCs promoted osteogenic capacity of older MSCs and enhanced new bone formation in older rats.^67^ In addition, EPC-derived exosomes accelerated bone regeneration during distraction osteogenesis by stimulating angiogenesis.^68^

As a major cargo of exosomes (Section 2.1), RNA can alter recipient cell gene expression and phenotypic function, with microRNA (miRNA) and long non-coding RNA (lncRNA) being the most widely studied.^69^ From the perspective of an exosomal miRNA, one group discovered that miR-136-5p from bone marrow MSC-derived exosomes promoted osteoblast proliferation and differentiation in vitro, thereby promoting fracture healing in vivo.^70^ This was achieved by inhibiting the downstream target gene of miR-163-5p, low-density lipoprotein receptor-related protein 4 (LRP4), through the Wnt/β-catenin pathway. The other group found that MSC-derived exosomal miR-25 could regulate the ubiquitination and degradation of Runt-related transcription factor 2 (Runx2) by Smad ubiquitination regulatory factor 1 (SMURF1) to promote fracture healing in mice.^71^ From a lncRNA perspective, especially the bone-specific lncRNA H19, a Chinese group revealed that although a high-fat diet reduced osteogenic differentiation and weakened fracture healing, this could be reversed by MSC-derived exosomal lncRNA H19 via miR-467/HoxA10 axis in an obesity-induced fracture model.^72^ In addition, an American group demonstrated that exosomal lncRNA H19 not only improved osteogenesis but also angiogenesis through the angiopoietin 1/Tie2-NO signaling pathway in an immunocompromised nude mouse model.^73^

Instead of using naturally derived exosomes from MSCs, some researchers have conducted pre-isolation modification of exosomes to achieve better results. Liang et al. preconditioned MSCs with low doses of dimethyloxaloylglycine (DMOG), a small angiogenic molecule, to prepare the exosomes for an enhanced angiogenesis and bone regeneration in a critical-sized calvarial defect model by targeting the protein kinase B/mechanistic target of rapamycin (Akt/mTOR) pathway.^74^ Alternatively, Lu et al. loaded MSC-derived exosomes with miR-29a, which showed a robust ability in promoting angiogenesis and osteogenesis by targeting vasohibin 1.^75^ Furthermore, umbilical cord MSC-derived exosomes demonstrated comparable results to their bone marrow counterparts during fracture healing.^76^ In addition, exosomes derived from MSCs under hypoxia exhibited better effects on bone fracture healing than those under normoxia. Mechanistically, hypoxia preconditioning enhanced the production of exosomal miR-126 through the activation of hypoxia-inducible factor 1 (HIF-1α). Various studies have shown that hypoxia preconditioning represents an effective and promising optimization of the therapeutic effects of MSC-derived exosomes for bone fracture healing.

### Osteoarthritis

Osteoarthritis (OA) is the most common joint disease and most frequent reason for activity limitation in adults, affecting approximately 240 million patients globally.^77^ The pathology of OA has evolved from being viewed as cartilage-only to a multi-tissue disease that affects all components of the whole joint, including bone, synovium, muscle, ligament, and periarticular fat.^78^ Clinical trials have successfully revealed systemic compounds that arrest structural progression (e.g., cathepsin K and Wnt inhibitors) or reduce OA pain (e.g., nerve growth factor inhibitors). As a potential treatment option for OA, most MSC-derived exosome therapy used chondrocytes as a target in in vitro models. These MSCs could originate from various tissues, such as bone marrow, synovium, gingiva, and infrapatellar fat pads (IPFPs).

Some studies focusing on chondrogenesis demonstrated a particular interest in the role of miRNA. Wu et al. found that IPFP MSC-derived exosomes protect articular cartilage from damage and ameliorate gait abnormality in OA mice by miR100-5p-regulated inhibition of mTOR-autophagy pathway.^79^ Since it is easy to retrieve human IPFP from OA patients by arthroscopic operation within a clinic, this type of exosome therapy might simplify and accelerate the process from bench to bedside. Liu et al. discovered that MSC-derived exosomes could promote proliferation and inhibit apoptosis of chondrocytes via lncRNA-KLF3-AS1/miR-206/GIT1 axis in OA.^80^ The cellular work conducted by Kong et al. showed that synovial MSC-derived exosomal miR-320c could enhance chondrogenesis by targeting ADAM19.^81^ In addition, Mao et al. suggested that exosomal miR-92a-3p from chondrogenic MSCs could enhance chondrogenesis and suppress cartilage degradation via targeting Wnt5a.^82^ In contrast to these studies using original exosomes, few groups modified exosomes prior to their systemic administration. Tao et al. modified exosomes by transfecting synovial MSCs with miR-140-5p and found that exosomal miR-140-5p-overexpression could enhance cartilage tissue regeneration and prevent OA of the knee in a rat model.^83^ Meanwhile, Wang et al. used TGF-β1 to stimulate MSCs, and the resultant exosomal miR-135b increased chondrocyte proliferation by regulating specificity protein-1.^84^ In a comparative study, Zhu et al. demonstrated that exosomes from iPSC-derived MSCs could provide a stronger therapeutic effect on OA than synovial membrane MSC-derived exosomes.^85^

Other studies have focused on not only chondrogenesis but also anti-inflammation and immune modulation during OA treatment. For example, MSC-derived exosomes inhibited inflammatory factors, glutamine metabolic activity-related proteins, glutamine, and GSH/GSSG ratio in vitro, while improving mice’s chondrocyte function, tissue inflammation, and exercise capacity in vivo, thereby alleviating OA progression.^86^ Using a holistic approach, recent studies have shifted the attention away from cartilage towards other tissues (e.g., bone) in a diarthrodial joint. Firstly, bone marrow MSC-derived exosomal miR-206 promoted proliferation and osteogenic differentiation of osteoblasts in OA by reducing E74-like factor 3 (Elf 3), and ameliorated inflammation and increased expression of osteocalcin and BMP2 in mouse femoral tissues.^87^ Secondly, MSC exosome-treated osteochondral defects demonstrated a regenerative immune phenotype, characterized by a higher infiltration of CD163^+^ M2 macrophages over CD86^+^ M1 macrophages, with a concomitant reduction in pro-inflammatory synovial cytokines IL-1β and TNF-α.^88^ Lastly, gingival MSC-derived exosomes proved to be immunosuppressive in preventing collagen-induced arthritis.^89^ Compared with parental cells, these exosomes had the same or stronger effects in inhibiting IL-17A and promoting IL-10, reducing incidences and bone erosion by arthritis, via inhibiting the IL-17RA-Act1-TRAF6-NF-κB signaling pathway.

Currently, there is no single ‘one size fits all’ drug that may be suitable for all OA patients. Disease-modifying OA drugs (DMOADs) might become the next-generation OA treatment.^90^ It is very valuable and relevant that MSC-derived exosome therapy for OA coincides with DMOADs: both are capable of targeting inflammatory cytokines, matrix-degrading enzymes, and the Wnt pathway. Thus, emerging approaches for DMOAD development, such as miRNA-based modality and targeting cellular senescence, might also be used to refine MSC-based exosome therapy for OA.

### Spinal cord injury

Traumatic spinal cord injury (SCI) is a devastating global health issue that poses a significant functional and economic burden both on the patient and society.^91^ The pathophysiology of SCI includes primary injuries caused by mechanical trauma and secondary injury cascade characterized by apoptosis, edema, ischemia, inflammatory cell infiltration, and excitotoxicity.^92^ Despite surgical intervention, clinical studies involving pharmacotherapy can be broadly classified as either neuroprotective or neuroregenerative.^93^ Targeting each event of the above mechanistic chain, both MSC- and NSC-derived exosome therapy could exert a beneficial influence on spinal cord protection and regeneration.

Some groups have targeted neuronal cell death. Ma et al. revealed that insulin-like growth factor 1 (IGF-1)-stimulated NSC-derived exosomes could inhibit neuronal apoptosis while promoting functional recovery after SCI through a miR-219a-2-3p/YY1 pathway.^94^ Alternatively, Zhang et al. discovered that subarachnoid injection of NSC-derived exosomes could suppress neuronal cell apoptosis by activating autophagy via miR-374-5p/STK-4 axis for enhanced functional recovery in SCI.^95^ Shao et al. explored other forms of cell death (e.g., ferroptosis) using MSC-derived exosomes, and found that exosomal lncGm36569 could inhibit neuronal cell ferroptosis via miR-5627-5p/FSP1 axis, thereby decreasing neuronal dysfunction.^96^

Some groups have targeted anti-inflammation and immunomodulation. Nakazaki et al. discovered that fractionated intravenous infusion of MSC-derived exosomes could target M2 macrophages and upregulate TGF-β, thereby stabilizing microvessels and improving functional recovery.^97^ Similarly, Liu et al. demonstrated that in addition to hypoxia increasing exosome production from bone marrow MSCs, preconditioned exosomal miR-216a-5p could also repair traumatic SCI by shifting microglial M1/M2 polarization via the TLR4/NF-κB/PI3K/Akt pathway.^98^ Huang et al. valuably proved that epidural fat MSC-derived exosomes could attenuate NLRP3 inflammasome and improve functional recovery in SCI.^99^ Compared to MSC-derived exosomes, exosomes derived from EPCs could provide comparable anti-inflammatory effect. Yuan et al. showed that the exosomal miR-222-3p from EPCs could promote anti-inflammatory macrophages via the SOC3/JAK2/STAT3 pathway and improve mouse functional repair after SCI.^100^

Some groups have targeted angiogenesis and blood-spinal cord barrier (BSCB) integrity. For example, Zhong et al. used unmodified NSC-derived exosomes, found that they were highly enriched in VEGF-A, and could therefore enhance the angiogenic activity of spinal cord microvascular endothelial cells (SCMECs).^101^ In comparison, Chen and co-workers modified NSC-derived exosomes with FTY720, an immune modulator and microvascular regulator, to protect the barrier function of SCMECs via the PTEN/Akt pathway, thereby ameliorating hindlimb function.^102^ It is well-known that the connection between the microvascular endothelium of the spinal cord and the pericyte is crucial in maintaining the structural integrity of BSCB.^103^ Thus, Zhou’s team attempted to verify the role of exosome therapy in pericyte homeostasis.^104^ They proved that bone marrow MSC-derived exosomes could reduce pericyte pyroptosis and increase pericyte survival rate in vitro, while improving BSCB integrity and locomotor recovery in vivo.

Finally, some groups have targeted other aspects during neuroprotection and neuroregeneration, such as neurotoxic astrocytes and endogenous NSC sustainability. Lai et al. proved that human umbilical cord MSC-derived exosomes could facilitate recovery of spinal cord function by targeting neurotoxic astrocytes.^105^ In addition, miR-146a-5p-modified exosomes exerted a more powerful effect than unmodified exosomes. Li et al. discovered that exosomes derived from nerve growth factor (NGF)-overexpressing bone marrow MSCs could enhance neuronal differentiation of NSCs and axonal regeneration.^106^ Zhou et al. demonstrated that placental MSC-derived exosomes could promote the activation of proliferating endogenous NSCs, thereby improving both locomotor activity and bladder dysfunction,^107^ which is a frequent sequelae that could further worsen the quality of life of SCI patients.^108^

### Muscle and tendon tear

Muscle and tendon tears can result from either acute trauma (e.g., fractures, Section 3.1) or chronic overuse (e.g., sports injury).^109^ Healing of muscle strain and tendon tear follows the typical wound healing course, involving the inflammatory, proliferative, and remodeling phases. Multiple non-surgical strategies have been trialed to improve healing, including cell-based and growth factor-based therapies.^110^ The following proof-of-concept studies indicate that MSC-derived exosomes could become the next-generation musculoskeletal treatment.

On the one hand, some groups have focused on individual components of the muscle-tendon-bone unit. Nakamura et al. claimed that MSC-derived exosomes could improve in vitro myogenesis in C2C12 myoblasts and angiogenesis in HUVECs, while accelerating in vivo skeletal muscle regeneration in a cardiotoxin-induced muscle injury model.^111^ These benefits were at least in part mediated by miRNAs such as miR-494. Chen et al. discovered that exosomes from adipose MSCs could enhance the proliferation and the migration of primary tenocytes, while also improving mechanical strength of repaired tendons by upregulating decorin and biglycan in a rabbit Achilles tendon rupture model.^112^

On the other hand, some groups have regarded the muscle-tendon-bone unit as a single functional system and used rotator cuff tear as the disease model, which is the most common shoulder condition for which patients seek treatment.^113^ One group of researchers published two consecutive studies using adipose MSC-derived exosomes. In a rat model of massive rotator cuff tear, exosome therapy could prevent the atrophy, inflammation, and vascularization of muscles.^114^ In a rabbit model of chronic rotator cuff tear, exosome therapy could prevent fatty infiltration and improve biomechanical properties.^115^ Another group reported that bone marrow MSC-derived exosomes could increase the breaking load and stiffness of the rotator cuff after reconstruction, induce angiogenesis around the rotator cuff endpoint, and promote growth of the tendon-bone interface.^116^

### Other orthopedic diseases

Osteoporosis is a metabolic bone disease characterized by low bone density and weakening of bone architecture, which increase the risk of fractures. It results from osteoclastic bone resorption undercompensated by osteoblastic bone formation.^117^ In a cellular study, adipose MSC-derived exosomes could antagonize hypoxia/serum deprivation-induced osteocyte apoptosis and osteocyte-mediated osteoclastogenesis.^118^ Further animal studies revealed that umbilical cord MSC-derived exosomes could inhibit bone marrow MSC apoptosis and prevent disuse osteoporosis via miR-1263/Mob1/Hippo pathway,^119^ and improve tibial density and reverse estrogen-deficient osteoporosis via miR-2110 and miR-328-3p.^120^

Compared to SCI, damage to peripheral nerve (e.g., sciatic nerve injury) is considerably more common. The subsequent nerve regeneration is controlled by the interplay between neurons and Schwann cells, and further complicated by inflammatory cell infiltration.^121^ It was shown that adipose MSC-derived exosomes could target neurons by increasing neurite outgrowth in vitro and axonal regeneration and walking behavior in vivo.^122^ Adipose MSC-derived exosomes could target Schwann cells by promoting the proliferation, migration and secretion of neurotrophic factors in vitro and restore denervation muscle atrophy in vivo.^123^ LPS-preconditioned MSC-derived exosomes could target inflammatory cells by enhancing M2 macrophage polarization in vitro and accelerate peripheral nerve regeneration in vivo.^124^

Intervertebral disc (IVD) degeneration is a major cause of lower back pain which is the leading injury in total global years lived with disability. Its molecular processes include extracellular matrix (ECM) degeneration, inflammation, oxidative stress, apoptosis, senescence and reduced autophagy.^125^ The emerging avenues of exosome therapy attempt to solve some of these issues. Cheng et al. demonstrated that intradiscal injection of bone marrow MSC-derived exosomes could inhibit nucleus pulposus cell (NPC) apoptosis and alleviate IVD degeneration via exosomal miR-21.^126^ On the other hand, Chen et al. discovered that human ESC-derived exosomes could inhibit NLRP3 inflammasome to alleviate pyroptosis in nucleus pulposus cells by delivering miR-302c.^127^ In addition to cell death and mitochondrial damage, oxidative stress in NPCs was also found to be inhibited by MSC-derived exosomes.^128^ Since IVD degeneration and OA share a common molecular disease spectrum^125^ the positive results of OA treatment using MSC-derived exosomes (Section 3.2) could be used as a reference for IVD degeneration research.

Osteonecrosis, aka., avascular necrosis, of the femoral head (ONFH) is a disabling condition affecting a younger population, which often results in total hip arthroplasty.^129^ Glucocorticoid (GC)-induced osteonecrosis is one of the most common causes of ONFH, whose pathogenesis is manifested in two aspects: compromised blood supply to the femoral head and dampened osteogenic activity. Liu et al. showed that exosomes from iPSC-derived MSCs could prevent GC-induced ONFH by promoting angiogenesis and osteogenesis via the PI3K/Akt pathway.^130^ Zuo et al. demonstrated that miR-26a-overexpressing exosomes derived from HSCs could provide similar therapeutic effects.^131^

## Neurosurgery and SC-Exo therapy

### Ischemic stroke

Strokes are the second highest cause of death and the third leading cause of disability globally, with ischemic stroke being the most common subtype.^132^ The key events during the ischemic cascade include neuronal dysfunction, excitotoxicity, neurochemical injury, and neuroinflammation.^133^ In terms of treatment, a new generation of clinical trials is now underway, which uses cytoprotective drugs, such as immunomodulators, IL-6 receptor antagonists, Rho kinase inhibitors, and free radical scavengers.^134^ Targeting each event of the above pathophysiology, nearly all subtypes of SC-exo demonstrated potent therapeutic effects on stroke recovery (Table 3).

**Table 3:** Stem cell-derived exosomes for the treatment of diseases in neurosurgery and related specialties

Target disease	Exosome	In vitro model & findings	In vivo model & findings	Refs.
Ischemic stroke	NSC-exo	SH-SY5Y cells; inhibited apoptosis and promoted proliferation both in normal and OGD conditions	rat MCAO model; reduced infarction area and neuron apoptosis, exosomal miR-150-3p enhanced neuroprotective effects by targeting CASP2	^135^
	EPC-exo	N/A	rat MCAO model; reduced infarct size, neurological defect score, and percentage of apoptotic cells, but increased CD31 and VEGF	^137^
	IFN-γ induced NSC-exo	NSCs; increased cell proliferation & survival, and reduced cell apoptosis	rat MCAO model; promoted behavioral and structural outcomes; inflammatory factor IFN-γ preconditioned exo were more potent	^138^
	BM-MSC-exo	OGD N2a cells, rat primary cortical neurons; neuroprotective against NLRP3 inflammasome-mediated pyroptosis	N/A	^139^
	iPSC-exo	rat primary cortical neurons; improved neuronal survival and neurite outgrowth via PTEN/Akt pathway	N/A	^140^
	NSC-exo, iCM-exo	primary mouse cortical astrocytes, neuronal cells; protected after OGD ischemia; NSC-exo > iCM-exo	mice; reduced infarct volume	^141^
	Zeb2/Axin2 enriched BM-MSC-exo	OGD rat neuron; increased neurite branching & elongation	rat MCAO model; improved post-stroke neurogenesis, neural plasticity, and spatial memory and nerve function, likely via SOX10, Wnt/β-catenin, and endothelin-3/EDNRB pathways	^142^
	miR-126-EPC-exo	N/A	diabetic mouse MCAO model; improved acute brain injury and functional recovery after stroke by promoting neurogenesis	^143^
	BM-MSC-exo	BV2 microglia; induced microglia deactivation and M2 polarization	rat MCAO model; reduced infarct size and improved neuronal function via transferring miR-23a-3p	^144^
	UC-MSC-exo	BV2 microglia; attenuated microglia-mediated inflammation after OGD	mice; reduced infarct volume, behavioral deficits, and ameliorated microglia activation; exosomal miR-146a-5p reduced neuroinflammation via IRAK1/TRAF6 pathway	^145^
	TSG101-oe-NSC-exo	N2A cells; attenuated LDH release and proinflammatory factors,	rat MCAO model; reduced infarction volume & inflammatory cytokines, inhibited DNA-damage pathway, and upregulated neurotrophic factors	^146^
	RGD NSC-exo	ReN & BV2 cells; showed intrinsic anti-inflammatory activity	mice; targeted ischemic brain regions and suppressed postischemia inflammatory response; exosomal miRs inhibited MAPK pathway	^147^
	BDNF-NSC-exo	H_2_O_2_-induced oxidative stress in NSCs; reduced apoptosis and increased neurogenic differentiation	rat MCAO model; inhibited the activation of microglia, promoted the differentiation of endogenous NSCs into neurons, and improved behavioral function	^149^
	miR-210-EPC-exo	H/R injured SH-SY5Y cells; protected from apoptosis & oxidative stress	N/A	^150^
	ACE2-enriched EPC-exo	H/R injured mouse brain microvascular endothelial cells; inhibited senescence	mouse MCAO model; exosomal miR-17-5p inhibited apoptosis, oxidative stress & brain dysfunction via PTEN/PI3K/Akt pathway	^152^
	NSC-exo + EPC-exo	H/R injured SH-SY5Y cells; protected from apoptosis & oxidative stress	rat MCAO model; reduced infarct volume & neurological deficits score via Nox2/ROS & BDNF/TrkB pathways	^153^
Traumatic brain injury	MSC-exo	N/A	rats; improved spatial learning & sensorimotor function and neurovascular plasticity	^159^
	BM-MSC-exo	N/A	rats; improved spatial learning, and 3D > 2D culture conditions; enhanced sensorimotor recovery; increased endothelial cells & neurons, and reduced neuroinflammation	^160^
	MSC-exo	N/A	primary motor cortex monkey model; animals returned to pre-operative grasp patterns & latency to retrieve food reward in the first 3–5 weeks of recovery	^161^
	MSC-exo	N/A	combined TBI & HS swine model; attenuated severity of neurologic injury and allowed for faster neurologic recovery	^162^
	adipose MSC-exo	primary rat microglia & neuron; suppressed microglia activation by inhibiting NF-κB & MAPK	rats; promoted functional recovery, suppressed neuroinflammation, reduced neuronal apoptosis, and increased neurogenesis; exo mainly taken up by microglia/macrophages	^163^
	BM-MSC-exo	BV2 microglia; promoted M1 to M2 phenotype and upregulated anti-inflammatory cytokines	mice; reduced cortical tissue apoptosis and inhibited neuroinflammation, possibly by exosomal miR-181b via IL-10/STAT3 pathway	^164^
	NSC-exo	NSCs; exo superior to parental cells	rats; improved neurobehavioral performance, inhibited astrocyte neuroinflammation, enhanced doublecortin neurogenesis, while maintaining SOX2 & Nestin stemness	^165^
	MSC-exo	N/A	rats; improved angiogenesis & neurogenesis, and sensorimotor & cognitive function, reduced neuroinflammation & hippocampal neuronal cell loss; 100 µg & 1 day were optimal	^166^
Alzheimer’s disease	BM-MSC-exo	N/A	early-stage AD mice; reduced Aβ plaque burden & dystrophic neurites; carried neprilysin	^179^
	BM-MSC-exo	primary neuron; reduced Aβ-induced iNOS expression	mice; rescued synaptic impairment and improved cognitive behavior	^180^
	NSC-exo	N/A	AD transgenic mice; enhanced mitochondrial function, sirtuin 1 activation, synaptic activity, decreased inflammatory response, and rescued cognitive deficits	^181^
	heat shock-induced NSC-exo	HC2S2 cells; exhibited greater neuroprotection against oxidative stress and Aβ-induced neurotoxicity	N/A	^182^
	MSC-exo	SH-SY5Y with FAD mutations; reduced Aβ expression and restored neuronal memory	AD transgenic mice; improved brain glucose metabolism and cognitive function; upregulated synapse-related genes & downregulated HDAC4 expression	^183^
	AF-MSC-exo	BV2 microglia, SH-SY5Y cells; mitigated neuroinflammatory microglial injury and recovered neurotoxicity from Aβ	N/A	^184^
	UC-MSC-exo	BV2 microglia; reduced inflammatory reaction & induced alternative microglial activation	mice; alleviated neuroinflammation and reduced Aβ deposition by modulating microglial activation; increased spatial learning & memory function	^185^
	MSC-exo	N/A	mice; stimulated neurogenesis in subventricular zone and alleviated Aβ-induced cognitive impairment	^186^
	NSC-exo	5xFAD primary cerebral endothelial cells; reversed AD-caused BBB deficiency	5xFAD mouse model; BBB breakdown occurred at 4 months of age, which could be mimicked with an in vitro BBB model	^188^
	RVG-tagged MSC-exo	N/A	transgenic APP/PS1 mice; improved CNS-targeted delivery; reduced Aβ deposition & astrocytes, and improved cognitive function; RVG-exo were better	^189^
Parkinson’s disease	BM-MSC-exo	SH-SY5Y & SK-N-SH cells; exosomal TSG-6 attenuated MPP^+^-induced neurotoxicity via STAT3/miR-7/NEDD4 axis	N/A	^193^
	NSC-exo	SH-SY5Y & BV2 cells; anti-oxidative stress, anti-inflammatory & anti-apoptotic effects	6-hydroxydopamine-induced PD mice; protected dopaminergic cell viability via exosomal miR-182-5p, miR-183-5p, & miR-9	^194^
Multiple sclerosis	BM-MSC-exo	HAPI microglia; downregulated TNF-α & iNOS and upregulated IL-10, TGF-β and arginase-1	EAE rat model; reduced inflammation and demyelination of CNS by regulating polarization of microglia from M1 to M2; decreased neurobehavioral scores and prevented weight loss	^197^
	BM-MSC-exo	N/A	2 mice models: EAE & CPZ; improved neurological outcome, increased OPC differentiation & remyelination, decreased neuroinflammation via TLR2 pathway	^198^
Vascular dementia	NSC-exo	N/A	rats; exo-derived MIAT improved learning ability & memory via miR-34b-5p/calbindin-1 axis	^199^
HIV-associated neurocognitive disorders	NSC-exo	rescued cellular viability in HIV-damaged neurons, and inhibited apoptosis and inflammatory factor secretion	N/A	^200^
Radiation-induced cognitive dysfunction	NSC-exo	N/A	mice; exosomal miR-124 improved exercise & fear behavior, reversed cognitive impairment, and reduced neuroinflammation	^201^
	NSC-exo	N/A	mice; protected host neurons, enhanced neurotrophic factors & synaptic signaling, and reduced neuroinflammation	^202^
Epilepsy	BM-MSC-exo	N/A	mice; reduced hippocampal inflammation, and prevented abnormal neurogenesis & memory dysfunction	^203^
Mechanical allodynia	BM-MSC-exo	microglia; downregulated NOTCH2 which is targeted by exosomal miR-150-5p	L5 spinal nerve ligation rat model; increased paw withdrawal threshold and latency, reduced apoptosis and inflammation in spinal dorsal horn	^204^
Spina bifida aperta	NSC-exo	BM-MSCs; promoted neuronal differentiation of MSCs	rat embryo model; exosomal Netrin 1 promoted neuronal differentiation of MSCs & NSCs by upregulating Hand2/Phox2b	^205^
Depression	BM-MSc-exo	N/A	rats; suppressed apoptosis & boosted proliferation in hippocampal tissues by upregulating exosomal miR-26a	^206^
Stress	UC-MSc-exo	N/A	mice acute brain disorder model; increased adiponectin, improved cognitive function and hippocampal neurogenesis that was suppressed by streptozotocin injection	^207^
Brain ageing	NSC-exo	NSCs; rescued IRS-1/FoxO activation and counteracted reduced proliferation and senescence	mice; intranasal administration counteracted HFD-dependent impairment of adult hippocampal neurogenesis by restoring balance between proliferating and senescent NSCs	^208^
	NSC-exo	NSCs; HFD downregulated CREB/BDNF/TrkB signaling	mice; intranasal administration restored CREB transcriptional activity, rescued both BDNF & HFD-dependent memory deficits	^209^
	hypothalamic NSC-exo	N/A	NSC-alation-induced mouse model; exosomal miRNAs reduced hypothalamic inflammation, and slowed down ageing, independent of food intake	^210^
Deep hypothermic circulatory arrest	MSC-exo	primary rat brain endothelial cells; rescued OGD-induced injury & inhibited TLR4/NLRP3/caspase-1/ NF-κB pathway	N/A	^211^

*Aβ* amyloid beta, *AD* Alzheimer’s disease, *AF* amniotic fluid, *BBB* blood brain barrier, *BDNF* brain-derived neurotrophic factor, *BM* bone marrow, *CREB* cAMP response element binding, *CNS* central nervous system, *EAE* experimental autoimmune encephalomyelitis, *EDNRB* endothelin receptor type B, *exo* exosome, *FAD* familial Alzheimer’s disease, *FoxO* Forkhead box O, *H/R* hypoxia and reoxygenation, *HAPI* highly aggressive proliferating immortalized, *HDAC* histone deacetylase, *HFD* high fat diet, *HS* hemorrhagic shock, *iCM* induced pluripotent stem cell-derived cardiomyocyte, *IFN* interferon, *IL* interleukin, *iNOS* inducible nitric oxide synthase, *iPSC* induced pluripotent stem cell, *IRAK* interleukin 1 receptor associated kinase, *IRS* insulin receptor substrate, *LDH* lactate dehydrogenase, *MAPK* mitogen-activated protein kinase, *MCAO* middle cerebral artery occlusion, *MIAT* myocardial infarction associated transcript, *miR* microRNA, *MPP*^+^ 1-methyl-4-phenylpyridinium, *MSC* mesenchymal stem cell, *NEDD4* neuronally expressed developmentally down-regulated 4, *NF-κB* nuclear factor-kappa B, *NLRP* NOD-, LRR- and pyrin domain-containing protein, *NSC* neural stem cell, *oe* overexpressing, *OGD* oxygen- & glucose-deprived, *SOX* Sry-Box transcription factor, *STAT* signal transducer and activator of transcription, *TGF* transforming growth factor, *TLR* Toll-like receptor, *TNF* tumor necrosis factor, *TRAF* TNF receptor associated factor, *TrkB* tropomyosin receptor kinase B, *TSG* TNF stimulated gene, *UC* umbilical cord, *Zeb* zinc finger E-box binding homeobox

Some groups have targeted neuroprotection and neurogenesis. Firstly, SC-exo therapy could inhibit neuronal cell death. Luo et al. found that NSC-derived exosomes could inhibit apoptosis while promoting the proliferation of SH-SY5Y cells both under normal and oxygen-glucose deprivation (OGD) conditions.^135^ This was also tested in a middle cerebral artery occlusion (MCAO) model as a reduced infarction area and neuronal apoptosis via exosomal miR-150-3p. Other in vitro and in vivo studies showed similar anti-apoptotic effects using EPC-derived exosomes.^136,137^ Zhang et al. discovered that the exosomal anti-apoptotic effect could be improved by preconditioning the parental NSCs with interferon gamma (IFN-γ).^138^ Secondly, SC-exo therapy could protect cells of the CNS. Kang et al. revealed that exosomes derived from bone marrow MSCs could rescue OGD-induced injury in neural cells by suppressing NLRP3 inflammasome-mediated pyroptosis.^139^ Exosomes sourced from hypoxic cultures had a more pronounced neuroprotective effect than their counterparts from normal cultures. Similarly, Li et al. discovered that exosomes derived from human iPSC-derived neural progenitor cells exhibited a neuroprotective effect on OGD neurons and neurite outgrowth.^140^ This protection of neuronal function under ischemic conditions was regulated through the PTEN/Akt pathway. In addition, Sun et al. proved that NSC-derived exosomes could also protect astrocytes, which become supporting reactive astrocytes (RAs) after strokes.^141^ Thirdly, SC-exo therapy could improve post-stroke neurogenesis. Wei et al. suggested that Zeb2/Axin2 from bone marrow MSC-derived exosomes could improve post-stroke neurogenesis, neural plasticity, and spatial memory and nerve function, likely via the SOX10, Wnt/β-catenin, and endothelin-3/EDNRB pathways.^142^ Wang et al. illustrated that miR-126-modified EPC-derived exosomes could alleviate acute brain injury and promote functional recovery after stroke by enhancing neurogenesis.^143^

Some groups have targeted the inhibition of the neuroinflammation. Firstly, unmodified SC-exo therapy exhibited an anti-inflammatory effect through exosomal miRNAs. Dong et al. showed that bone marrow MSC-derived exosomes could induce BV2 microglia deactivation and M2 polarization in vitro, while reducing infarct size and improving neuronal function in vivo via transferring miR-23a-3p.^144^ Similarly, Zhang et al. unveiled that umbilical cord MSC-derived exosomal miR-146a-5p could attenuate microglia-mediated neuroinflammation after OGD in vitro, while improving behavioral deficits and microglia activation in vivo via the IRAK1/TRAF6 signaling pathway.^145^ Secondly, the anti-inflammatory effect of SC-exo therapy could be enhanced by modifying the exosomes. Yoon and co-workers established tumor susceptibility gene (TSG)101-overexpressing human NSCs, thereby increasing exosome secretion.^146^ The engineered exosomes not only attenuated LDH release and proinflammatory factors in vitro, but also reduced infarction volume, inhibited DNA-damage pathway, and upregulated neurotrophic factors in vivo. Furthermore, Tian’s team broke new ground by ingeniously attaching RGD peptide onto an NSC-derived exosome membrane, which targeted the lesion region of the ischemic brain after intravenous administration, thereby suppressing the inflammatory response after cerebral ischemia by inhibiting the MAPK pathway.^147^ Interestingly, Gao et al. used induced NSCs (iNSCs) reprogrammed from mouse fibroblasts for stroke treatment. They showed that iNSC-derived exosomes, bearing similar therapeutic effects with NSC-derived ones, could not only promote neurogenesis but also inhibit neuroinflammation.^148^

Finally, some groups have targeted other aspects during stroke recovery, such as neurochemical injury and oxidative stress. Zhu et al. loaded brain-derived neurotrophic factor (BDNF) into exosomes derived from NSCs to construct engineered exosomes.^149^ In a model of H_2_O_2_-induced oxidative stress, exosome therapy significantly enhanced NSC survival. In a rat MCAO model, exosome therapy not only inhibited microglial activation, but also boosted the differentiation of endogenous NSCs into neurons. Collectively, BDNF-based modification of NSC-derived exosomes has improved effects in the treatment of ischemic stroke. On the other hand, miR-210-modified EPC-derived exosomes could protect neurons from hypoxia and reoxygenation (H/R)-induced apoptosis, oxidative stress, and decreased viability, thereby supporting the treatment of ischemic stroke.^150,151^ The exosomal miR-17-5p from ACE2-enriched EPC-derived exosomes could ameliorate cerebral ischemic injury in aged mice.^152^ In an intriguing study conducted by Xu and co-workers, combination of NSC-exo and EPC-exo with miR-210 and miR-123 overexpression exerted better therapeutic effects on ischemic stroke by protecting H/R injured neurons through the BDNF-TrkB and Nox2/ROS pathways.^153^

In contrast to ischemic stroke, hemorrhagic stroke poses a deadlier threat and worse disability in most survivors.^154^ miR-137 overexpression was found to boost the neuroprotective effects of EPC-derived exosomes against apoptosis, ferroptosis, and mitochondrial dysfunction in oxyhemoglobin-treated SH-SY5Y cells, an in vitro hemorrhagic stroke model, partially through the COX2/PGE2 pathway.^155^

### Traumatic brain injury

Approximately 70 million patients suffer from traumatic brain injury (TBI) globally each year, which poses serious physical, psychosocial and economic threats.^156^ TBI can be categorized as primary injuries (e.g., axonal death, neuroinflammation, neurochemical change, and metabolic dysfunction) and secondary injuries (e.g., ischemic and hypoxic damage, cerebral edema, raised intracranial pressure, hydrocephalus, and infection).^157^ Each patient with a TBI has a unique set of circumstances depending on variables such as the location and severity of the injury, making medical and surgical treatment quite challenging.^158^ Therefore, systemic therapy using SC-exo may become a ‘one-size-fits-all’ option for managing TBI.

A series of animal studies published initially focused on the functional recovery and macroscopic aspects of MSC-derived exosome therapy. In a rat TBI model, exosome-treated animals showed significant improvement in spatial learning and sensorimotor function.^159^ In addition, exosome treatment significantly increased the number of newborn endothelial cells in the lesion boundary zone, and newborn immature and mature neurons in the dentate gyrus. In another rat TBI model with similar findings, exosomes derived from MSCs cultured in a 3D system provided better outcomes than those in a conventional 2D condition.^160^ In a monkey model of TBI to the primary motor cortex, exosome-treated animals returned to pre-operative grasp patterns and latency to retrieve a food reward in the first 3–5 weeks of recovery.^161^ In an even more complicated and clinically realistic large animal model, in which both TBI and hemorrhagic shock were investigated, exosome therapy attenuated the severity of neurologic injury and enabled faster neurologic recovery.^162^

In comparison, studies completed in recent years shed new light on the molecular mechanisms underlying SC-exo therapy for TBI. Chen et al. reported that adipose MSC-derived exosomes could promote functional recovery, suppress neuroinflammation, reduce neuronal apoptosis, and increase neurogenesis. This was achieved through the uptake of exosomes specifically by microglia and suppression of their activation by inhibiting the NF-κB & MAPK pathways.^163^ Wen et al. showed that bone marrow MSC-derived exosomes could reduce cell apoptosis in cortical tissue of mouse models of TBI, inhibit neuroinflammation, and promote the transformation of microglia to the anti-inflammatory phenotype. This was realized by the action of miR-181b on the IL-10/STAT3 pathway.^164^ Abedi et al. proved that NSC-derived exosomes could improve neurobehavioral performance, inhibit astrocyte neuroinflammation, enhance neurogenesis, while maintaining NSC stemness.^165^ A valuable additional finding was that exosomes seemed to be superior to the parent NSCs in terms of sensorimotor functional recovery. Finally, a dose-response and therapeutic window demonstrated that MSC-derived exosomes could improve angiogenesis and neurogenesis, and sensorimotor and cognitive function, while reducing neuroinflammation and hippocampal neuronal cell loss.^166^ Although 100 µg and 1 day might be the optimal dose and therapeutic window respectively, exosomes exhibited a wide range of effective doses for treatment of TBI within a therapeutic window of at least 7 days post-injury.

TBI and SCI are two of the most severe CNS traumas, which are increasingly recognized as global health priorities. The emerging evidence presented in Sections 3.3 and 4.2 are mutually beneficial for these two closely related research subspecialties. Henceforth, future research on SC-exo therapy for TBI and SCI could be either mechanism-based (e.g., the role of brain-gut axis^167^ transcriptional factors^168^ inflammasome^169^ and the complement system^170^) or modification-based (loading exosomes with drugs, e.g., immunomodulators^171^ antioxidants^172^ circular RNAs^173^ and microRNAs^174^).

### Alzheimer’s disease

Unlike TBI and SCI, which are traumatic in nature, Alzheimer’s disease (AD) and Parkinson’s disease (PD) are the most common neurodegenerative diseases (NDD). The hallmarks of NDD include, but are not limited to, pathological protein aggregation, synaptic and neuronal network dysfunction, aberrant proteostasis, cytoskeletal abnormalities, altered energy homeostasis, DNA and RNA defects, inflammation, and neuronal cell death.^175^ AD is the most common form of dementia globally and accounts for 25 million cases.^176^ Currently, only two classes of drugs are approved for symptomatic AD treatment, including cholinesterase inhibitors and NMDA antagonists. Although several therapeutics are actively undergoing clinical trials, none of them are near curative for AD.^177^ The challenges of brain-drug delivery, e.g., the blood-brain barrier (BBB) and pharmacokinetic drawbacks, are very likely to be solved by nanosized exosomes, which are additionally packaged with potent biomolecules. Most SC-exo therapy involves amyloid-β (Aβ), which is positioned at the center of AD pathophysiology.^178^

The initial work focused on the clearance of aggregation of the pathological protein, Aβ peptide. The intracerebral injection of MSC-derived exosomes by Elia and co-workers reduced Aβ plaque burden and dystrophic neurites in both the cortex and hippocampus in the early stages of a preclinical model of AD.^179^ In addition, using immunoblotting, the authors confirmed the presence of Neprilysin, a neutral endopeptidase capable of Aβ degradation, in the exosome’s lysates and its mRNA.

Some teams have focused on relieving synaptic dysfunction and oxidative stress. Wang et al. found that MSC-derived exosomes could rescue synaptic impairment and improve cognitive behavior in APP/PS1 mice, while alleviating exogenous Aβ-induced inducible nitric oxide synthase (iNOS) expression.^180^ Instead of using MSC-derived exosomes, Li et al. administered NSC-derived exosomes and enhanced mitochondrial function, sirtuin 1 activation, synaptic activity, and rescued cognitive deficits.^181^ Using alternative methods, Huber et al. noticed that heat shock-induced exosomes derived from NSCs exhibited greater neuroprotection against oxidative stress as well as Aβ-induced neurotoxicity.^182^

Some teams have centered their research around energy homeostasis. Chen et al. found that MSC-derived exosomes could improve brain glucose metabolism and cognitive function in AD transgenic mice using ^18^F-FDG PET/CT imaging and NOR testing, respectively.^183^

Some teams have focused on microglial neuroinflammation. In Zavatti’s cellular study, it was found that amniotic fluid MSC-derived exosomes could mitigate neuroinflammatory microglial phenotype and recover neurotoxicity from Aβ using LPS-stimulated BV2 microglia and SH-SY5Y neuroblastoma cells as models, respectively.^184^ Ding et al. showed that umbilical cord MSC-derived exosomes could alleviate neuroinflammation and reduce Aβ deposition by modulating microglial activation, thereby increasing spatial learning and memory function in AD mice.^185^

Some teams have focused on neuronal cell death and neurogenesis hoping to counteract AD progression. Reza-Zaldivar and co-workers gave MSC-derived exosomes to AD mice and the SC-exo therapy stimulated neurogenesis in the subventricular zone and alleviated Aβ-induced cognitive impairment.^186^ These effects are comparable to those shown in the MSCs.

Some teams have focused on the BBB, the dysfunction of which leads to increased permeability, microbleeds, impaired glucose transport, and degeneration of pericytes and endothelial cells.^187^ Liu et al. indicated that BBB breakdown in 5xFAD (familial Alzheimer’s disease) mice occurred at 4 months of age, and more importantly, treatment with NSCs-derived exosomes reversed AD-caused BBB deficiency.^188^

Finally, some groups have focused on improving the technicality of SC-exo therapy for AD. When exosomes are injected intravenously, they could be tracked in other organs instead of the targeted regions in the brain. Cui et al. conjugated MSC-derived exosomes with CNS-specific rabies viral glycoprotein (RVG) to target them to the brain of transgenic AD mice.^189^ The modified exosomes not only exhibited increased delivery to the cortex and hippocampus, but also significantly improved learning and memory capabilities with reduced Aβ deposition. On the other hand, Gao et al. obtained iNSCs through somatic cell reprogramming, which opened a new window for sourcing therapeutic exosomes. They demonstrated that iNSC-derived exosomes, bearing comparable therapeutic effects with NSC-derived ones, could mitigate various AD phenotypes, e.g., cognitive function, Aβ deposition, neuroinflammation, and neuroregeneration, in a preclinical mouse model.^190^

### Parkinson’s disease

Parkinson’s disease is the second most common neurodegenerative disease among the elderly, affecting more than 6 million patients worldwide.^191^ PD is caused by the necrosis of dopaminergic neurons in the substantia nigra and the presence of protein inclusions named Lewy bodies. The molecular pathophysiology includes α-synuclein proteostasis, mitochondrial dysfunction, oxidative stress, calcium imbalance, and neuroinflammation.^192^

In a study using bone marrow MSC-derived exosomes, Huang et al. discovered that exosome-derived TNF-stimulated gene-6 (TSG-6) could attenuate 1-methyl-4-phenylpyridinium ion (MPP + , metabolite of a neurotoxin MPTP)-induced neurotoxicity. In this in vitro PD model using SH-SY5Y and SK-N-SH cells, the exosomal anti-PD progression effect was found to be mediated through the STAT3/miR-7/NEDD4 axis.^193^

In a study using NSC-derived exosomes, Lee et al. revealed that SC-exo therapy could help to prevent the neuropathology and progression of PD.^194^ Working in vitro on SH-SY5Y and BV2 cells, NSC-derived exosomes could reduce the intracellular reactive oxygen species (ROS) and associated apoptotic pathways. Working in vivo on 6-hydroxydopamine-induced PD mice, NSC-derived exosomes could downregulate pro-inflammatory factors and significantly reduce dopaminergic neuronal loss. The presence of NSC-specific microRNAs, such as miR-182-5p, miR-183-5p, miR-9 and let-7, was confirmed and found to be involved in cell differentiation, neurotrophic function, and immune modulation.

### Multiple sclerosis

Multiple sclerosis (MS) is the most common non-traumatic, neurodegenerative, and disabling CNS disease affecting young adults. The pathological hallmark of MS is the formation of demyelinating lesions in the brain and spinal cord, with an inflammatory and autoimmune involvement.^195^ Currently licensed disease-modifying therapies include interferon-based, immunomodulatory, immunosuppressive, and immune reconstitution drugs.^196^ A few preliminary studies have highlighted the potential of MSC-derived exosomes for MS treatment.

In an animal experiment using experimental autoimmune encephalomyelitis (EAE) rats, Li et al. showed that SC-exo therapy significantly decreased neural behavioral scores, reduced the infiltration of inflammatory cells into the CNS, and decreased demyelination.^197^ In addition, exosome treatment upregulated M2-related cytokines while downregulating M1-related ones by regulating the polarization of microglia.

In an animal study using two mouse models of demyelination (the EAE model and the cuprizone diet model), Zhang et al. found that SC-exo therapy could promote remyelination by acting both directly on oligodendrocyte (OL) progenitors and indirectly on microglia.^198^ MSC-derived exosomes could improve neurological outcomes, increase the numbers of newly generated and mature OLs, decrease Aβ precursor protein density, decrease neuroinflammation by shifting from M1 to M2 phenotype, and inhibit the TLR2/IRAK1/NF-κB pathway.

### Other neurosurgical and related diseases

The surgical potential of MSC- and NSC-derived exosome therapy in four major types of neurosurgical or neurological diseases has been thoroughly discussed above. In addition to vascular disruption-, trauma-, neurodegeneration-, and autoimmune-related disorders, other diseases have been proven suitable targets for SC-exo therapy recently (Table 3). These include, but are not limited to: 1, dementia, such as vascular dementia.^199^ HIV-associated neurocognitive disorders^200^ and radiation-induced cognitive dysfunction^201,202^; 2, functional disorders, such as epilepsy^203^ and mechanical allodynia^204^; 3, congenital abnormalities, such as spina bifida aperta^205^; 4, neuropsychological conditions, such as depression^206^ and stress^207^; 5, brain aging^208–210^; 6, iatrogenic brain problems, such as deep hypothermic circulatory arrest.^211^

## Plastic surgery and SC-Exo therapy

Wound healing occurs in all parts of the human body, with cutaneous wounds being the most common. The highest wound-related expenses were attributed to surgical wounds followed by diabetic ulcers.^212^ The overall but overlapping phases of wound healing include hemostasis, inflammation, angiogenesis, proliferation and remodeling, each of which is governed by distinct cell types and modulated by various signaling pathways.^213^ More than half of relevant work using SC-exo therapy to boost cutaneous wound healing is MSC-based (Table 4).

**Table 4:** Stem cell-derived exosomes for the treatment of diseases in plastic surgery and related specialties

Target disease	Exosome	In vitro model & findings	In vivo model & findings	Refs.
Wound healing	UC-MSC-exo	PBMCs; inhibited cell proliferation, promoted Treg transformation & formation of endothelial tube	eczema mouse model; accelerated wound closure with more new epidermis & dermis and less scar; reduced integral score of skin injury and no. of lymphocyte infiltration in skin	^214^
	adipose-MSC-exo	N/A	HDM-induced mouse model; reduced IgE, eosinophil & mast cell count, and downregulated inflammatory cytokines	^215^
	educated BM-MSC-exo	HUVECs; promoted angiogenesis via Akt/eNOS pathway	mice; promoted cutaneous wound healing	^216^
	atorvastatin-treated BM-MSC-exo	HUVECs; promoted proliferation, migration, tube formation, and VEGF level	streptozotocin-induced diabetic wound rat model; exosomal miR-221-3p facilitated wound repair by enhancing angiogenesis via Akt/eNOS pathway	^217^
	deferoxamine -stimulated BM-MSC-exo	HUVECs; activated the PI3K/Akt pathway via miR-126-mediated PTEN downregulation	streptozotocin-induced diabetic wound rat model; accelerated cutaneous wound healing by promoting angiogenesis	^218^
	EPC-exo	HMECs; enhanced proliferation, migration & tubule formation	streptozotocin-induced diabetic wound rat model; accelerated cutaneous wound healing by promoting angiogenesis via Erk1/2 pathway	^220^
	EPC-exo	N/A	streptozotocin-induced diabetic wound mouse model; exosomal miR-221-3p accelerated cutaneous wound healing via p53 pathway	^221^
	adipose-MSC-exo	HDFs; improved proliferation & migration	mice; promoted wound healing via PI3K/Akt signaling pathway	^222^
	adipose-MSC-exo	HaCaT cells; promoted proliferation & migration by activating Akt/HIF-1α pathway	mice; promoted wound healing, which was eliminated by inhibition of p‑Akt and HIF‑1α	^223^
	iPSC-exo, MSC-exo	HDFs, HaCaT cells; accelerated proliferation via ERK1/2 pathway	N/A	^224^
	MALAT1-adipose-MSC-exo	HaCaT cells & HDFs; promoted wound healing by miR-124 via Wnt/β-catenin pathway	N/A	^225^
	MSC-exo	BJ cells; promoted fibroblasts migration	rats; exosomal miR-135a promoted cutaneous wound healing by inhibiting LATS2 expression	^226^
	EPC-exo	HaCaT cells; promoted proliferation & migration, and inhibited apoptosis	diabetic mice; accelerated wound healing via downregulating PPARγ	^227^
	UC-MSC-exo	H_2_O_2_-treated HaCaT cells; increased proliferation & migration, and suppressed apoptosis	mice; attenuated full-thickness skin wounding by enhancing epidermal re-epithelialization and dermal angiogenesis via suppressing AIF nucleus translocation	^228^
	UC-MSC-exo	N/A	mice; suppressed myofibroblast differentiation by inhibiting TGF-β/Smad2 pathway during wound healing; miR-21, -23a, -125b, -145 responsible for preventing scar formation	^229^
	UC-MSC-exo	HDFs; suppressed dermal fibroblasts-myofibroblasts transition via TGF-β/Smad2/3 pathway	N/A	^230^
	adipose-MSC-exo	primary HDFs; stimulated proliferation, migration, and collagen synthesis	mice; exo recruited to wound area, and accelerated cutaneous wound healing; increased collagen I & III in early stage and inhibited collagen in late stage to reduce scar formation	^231^
	ESC-exo	HUVECs; ameliorated senescence, proliferation, and migration	D-galactose-induced aging mice; exosomal miR-200a accelerated wound closure and enhanced angiogenesis via Nrf2 activation	^232^
	ESC-exo	HDFs; inhibited cellular senescence via TGF-β receptor 2 pathway	mice; exosomal mmu-miR-291a-3p accelerated excisional skin wound healing process	^233^
Skin flap	adipose-MSC-exo	HUVECs; increased cell proliferation, migration with more cord-like structures	I/R injury rat model; increased flap survival & capillary density, and decreased inflammatory reaction & apoptosis; H_2_O_2_-conditioned exo were better	^234^
Craniofacial defect	SCAP-exo	HUVECs; improved angiogenic capacity and cell migration	mice; promoted craniofacial soft tissue regeneration by enhancing Cdc42-mediated vascularization	^235^
Scleroderma	UC-MSC-exo	N/A	mice; attenuated myofibroblast activation and collagen deposition in dermal fibrosis by downregulating the TGF-β/Smad signaling pathway	^236^
Leishmaniasis	UC-MSC-exo + Aloe-Emodin	L929 & J744 cells, artificial wound model; healed 72% wound in 24 h	L.major promastigotes & amastigotes; inhibited for 4–10 folds; combinations superior to exo alone	^237^
Alopecia	NSC-exo	dermal papilla cells; exosomal miR-100 promoted cell proliferation	depilation-induced mice hair regeneration model; promoted hair follicle growth by activating Wnt/β-catenin pathway	^238^

*AIF* apoptosis-inducing factor, *Akt* protein kinase B, *BM* bone marrow, *eNOS* endothelial nitric oxide synthase, *EPC* endothelial progenitor cell, *ESC* embryonic stem cell, *exo* exosome, *HDF* human dermal fibroblasts, *HDM* house dust mite, *HIF* hypoxia-inducible factor, *HMEC* human microvascular endothelial cell, *HUVEC* human umbilical vein endothelial cell, *I/R* ischemia-reperfusion, *LATS* large tumor suppressor, *MALAT* metastasis associated lung adenocarcinoma transcript, *miR* microRNA, *MSC* mesenchymal stem cell, *NSC* neural stem cell, *PBMC* peripheral blood mononuclear cell, *PI3K* phosphoinositide 3-kinase, *PTEN* phosphatase & tensin homolog, *SCAP* stem cells from apical papilla, *TGF* transforming growth factor, *Treg* regulatory T cell, *UC* umbilical cord, *VEGF* vascular endothelial growth factor

In the inflammatory stage, exosomes could inhibit the proliferation of peripheral blood mononuclear cells and promote the transformation of regulatory T cells in vitro, and reduce the number of lymphocytic infiltrations in the skin.^214^ In addition, exosomes could reduce IgE, eosinophil and mast cell count, and downregulate inflammatory cytokines.^215^ In the angiogenic stage, educated exosomes (e.g., atorvastatin and deferoxamine) could promote angiogenesis in diabetic wounds via the Akt/eNOS and PTEN/PI3K/Akt pathways.^216–218^ EPC-derived exosomes could accelerate cutaneous wound healing by promoting angiogenesis^219^ through the Erk1/2 pathway^220^ and p53 pathway.^221^ In the proliferative stage, stem cell-derived exosomes could promote the proliferation and migration of fibroblasts and keratinocytes. Some were achieved through the PI3K/Akt^222^ Akt/HIF-1α^223^ ERK1/2^224^ and Wnt/β-catenin^225^ pathways, while others through inhibition of LATS2^226^ PPARγ^227^ and AIF nucleus translocation^228^ In the final remodeling stage of wound healing, granulation tissue is replaced by permanent scar, during which abnormal wound healing might occur (e.g., keloids and hypertrophic scars). MSC-derived exosomes could suppress fibroblast-myofibroblast transition via the TGF-β/Smad2 pathway^229,230^ and increase collagen synthesis in early stage and reduce in late stage^231^ thereby reducing scar formation.

Furthermore, ESC-derived exosomes were found to exert similar therapeutic effect for wound healing to MSC-derived ones. Chen et al. used human ESC-derived exosomes to help healing of pressure ulcer.^232^ They noticed that exosomes could ameliorate endothelial senescence by activating Nrf2 and recover aging-related angiogenic dysfunction, thereby accelerating wound healing.^232^ In addition, Bae et al. revealed that the exosomal mmu-miR-291a-3p from ESCs could inhibit cellular senescence in human dermal fibroblasts through the TGF-β receptor 2 pathway, thereby accelerating the excisional skin wound healing process.^233^

In addition to wound healing, other plastic surgery-related diseases have been proven to be suitable targets for SC-exo therapy (Table 4). These include, but are not limited to: 1, skin grafting, such as skin flaps^234^; 2, tissue loss, such as craniofacial defect^235^; 3, autoimmune skin diseases, such as scleroderma^236^; 4, skin infections, such as leishmaniasis^237^; 5, hair transplantation, such as for alopecia^238^; 6, skin aging^239^

## General surgery and SC-Exo therapy

As a major subspecialty of general surgery, hepatobiliary surgery has attracted tremendous attention to SC-exo therapy. Firstly, acute liver injury (ALI)/acute liver failure (ALF) is a rare but challenging syndrome manifested by hepatic dysfunction, coagulopathy, encephalopathy, and multiorgan failure. About 60% of cases with ALF require and undergo orthotopic liver transplantation or result in death.^240^ In one study, Lin’s team focused on the cell death aspect of ALI, and found that MSC-derived exosomes could protect against ferroptosis via stabilization of SLC7A11 in carbon tetrachloride-induced ALI.^241^ Alternatively, Shao’s team focused on the pre-isolation modification of the exosomes, and revealed that exosomes derived from umbilical cord MSCs could ameliorate IL-6-induced ALI through exosomal miR-455-3p.^242^ Secondly, in contrast to ALI, liver fibrosis occurs when the liver sustains a chronic injury, which may progress into cirrhosis, liver failure, hepatocellular carcinoma, and even death.^243^ Ma et al. discovered that MSC-originated exosomal circDIDO1 could suppress hepatic stellate cell activation by miR-141-3p/PTEN/Akt pathway in human liver fibrosis.^244^ In addition, Wang et al. found that exosomes derived from 3D human ESC spheroids could attenuate hepatic stellate cell activation and inhibit liver fibrosis through inactivation of the Smad pathway by exosomal miR-6766-3p.^245^ For those with end-stage liver fibrosis needing a liver transplant, liver ischemia reperfusion injury (IRI) is a serious complication for graft dysfunction and organ rejection.^246^ Yang et al. demonstrated that bone marrow MSC-derived exosomes could relieve hepatic IRI, reduce hepatocyte apoptosis, and decrease liver enzyme levels by enhancing autophagy.^247^ Du et al. showed that exosomes from iPSC-derived MSCs could protect liver against hepatic IRI via activating sphingosine kinase and the sphingosine-1-phosphate pathway.^248^ Thirdly, non-alcoholic fatty liver disease (NAFLD) is known to adversely affect stroke recovery. Using a type 2 diabetes mellitus mouse model, Venkat et al. demonstrated that HSC-derived exosomes could simultaneously reduce liver dysfunction and improve neurological and cognitive function.^249^ Lastly, acute pancreatitis is an unpredictable and potentially lethal disease, the prognosis of which mainly depends on whether it develops into multiple organ dysfunction syndrome.^250^ Chen et al. revealed that exosomes from iPSC-derived MSCs could improve myocardial injury caused by severe acute pancreatitis through the Akt/Nrf2/HO-1 pathway.^251^

Peripheral artery disease affects 200 million patients worldwide and, in its most severe stage, can cause critical limb ischemia, subjecting patients to increased risk of cardiovascular events, amputation and death.^252^ As a cell-free therapy, placenta MSC-derived exosome infusion could enhance angiogenesis in a murine auricle ischemic injury model using laser Doppler blood flow analysis.^253^ Mechanistically, MSC-derived exosomes not only promote tube-like structure formation in vitro, but also mobilize endothelial cells into subcutaneous Matrigel plug in vivo, mainly through exosomal pro-angiogenic microRNAs, such as miR-30b.^254^ In addition, HSC-derived exosomes could repair ischemic hindlimb in mice by improving limb perfusion, capillary density, motor function and their amputation.^255^ This was most likely caused by internalization of exosomal miR-126-3p by endothelial cells relative to smooth muscle cells and fibroblasts. Human iPSC-derived exosomes demonstrated similar neoangiogenic effect through the exosomal miR-199b-5p.^256^ On the other hand, endovascular re-canalization is increasingly being used to reestablish blood flow to ischemic areas and restore tissue loss or gangrene for patients with peripheral artery disease.^257^ Three independent teams all proved that EPC-derived exosomes could promote vascular repair and accelerate reendothelialization in rat models of balloon-induced vascular injury by enhancing endothelial cell function.^258–260^ In addition, Kong et al. demonstrated similar protective effect of EPC-derived exosomes against balloon injury by inhibiting neo-intimal hyperplasia.^261^ This was achieved through promotion of reendothelialization and suppression of restenosis rather than through the direct inhibition of proliferation and migration of smooth muscle cells.

McCulloh and his co-workers published an intriguing study on the SC-exo therapy for necrotizing enterocolitis (NEC) which has an overall mortality of over 30% for premature infants requiring surgery.^262^ The authors compared the therapeutic effect of exosomes derived from four different types of stem cells, i.e., amniotic fluid MSCs, bone marrow MSCs, amniotic fluid NSCs and neonatal enteric NSCs.^263^ When injected at a concentration of at least 4 × 10^8^, all types of SC-exo were shown to reduce the incidence and severity of experimental NEC as effectively as their parental stem cells.

Sepsis is a deadly and potentially preventable complication in general surgery, in which microvascular dysfunction leads to multi-organ failure and mortality.^264^ Using a murine sepsis model by cecal ligation and puncture (CLP), Zhou and co-workers demonstrated that EPC-derived exosomes could improve sepsis outcome.^265^ This was manifested by reduced lung and renal vascular leakage, improved organ function, and increased survival through the exosomal miR-126-5p and miR-126-3p. Similarly, Liu et al. exhibited protective effect of EPC-derived exosomes on sepsis-induced organ damage and immune suppression by the exosomal miR-382-3p through the IκBα/NF-κB pathway.^266^

## Cardiothoracic surgery and SC-Exo therapy

The world’s leading mortality is ischemic heart disease (IHD) which is primarily caused by obstructive coronary atherosclerosis. The rupture of an atherosclerotic plaque is the most common trigger of acute arterial thrombosis causing myocardial infarction (MI). Prolonged oxygen deprivation to the myocardium can lead to cardiomyocyte death. Although timely reperfusion is essential, myocardial IRI might occur, thus mitigating the beneficial effects of reperfusion. Despite modern coronary reperfusion, the mortality and morbidity associated with the development of heart failure as a consequence of acute MI remain substantial, highlighting the importance of next-generation cardioprotective therapies, such as SC-exo.^267^

The work conducted by Xing et al. focused on atherosclerosis. The adipose MSC-derived exosomal miR-342-5p was shown to protect endothelial cells against atherosclerosis by targeting PPP1R12B in a H_2_O_2_-challenged HUVEC model.^268^ The work conducted by Peng et al. and Gao et al. focused on MI. The exosomal miR-25-3p from MSCs could alleviate MI by targeting pro-apoptotic proteins and EZH2 in an OGD cardiomyocytes model and left anterior descending artery ligation animal model.^269^ Similarly, human iPSC-derived exosomes could improve recovery from MI without increasing the frequency of arrhythmogenic complications in a swine model.^270^ The work conducted by Wen et al. and Santoso et al. focused on the death of cardiomyocytes. MSC-derived exosomes could ameliorate cardiomyocyte apoptosis in hypoxic conditions through miR-144 by targeting the PTEN/Akt pathway^271^ whereas iPSC-derived exosomes could regulate autophagy in hypoxic cardiomyocytes.^272^ Both Katsur et al. and Chen et al. focused on myocardial IRI. The former team found that exosomes derived from non-cardiomyocyte-related cells, i.e., CTX0E03 NSCs, could reduce infarct size while delaying cardiomyocyte mitochondrial permeability transition pore opening through the JAK/STAT pathway.^273^ The latter team discovered that MSC-derived exosomal miR-143-3p could suppress myocardial IRI by regulating autophagy via the CHK2-Beclin2 pathway.^274^ Finally, Chen and co-workers proved that bone marrow MSC-derived exosomes could attenuate cardiac hypertrophy and fibrosis in pressure overload-induced remodeling, thereby providing a promising potential treatment for heart failure.^275^

In comparison to MSC-derived exosomes, exosomes derived from ESCs exhibited comparable therapeutic effect for cardiac conditions. In terms of protection of cardiomyocytes, Khan et al. discovered that the ESC-derived exosomal miR-294 could improve cardiomyocyte survival, promote neovascularization and inhibit fibrosis after MI, thereby augmenting post-MI cardiac function.^276^ In addition, Tavakoli Dargagni et al. demonstrated that ESC-derived exosomes could alleviate doxorubicin-induced cardiotoxicity by inhibiting TLR4-NLRP3-mediated pyroptotic cell death in cardiomyocytes.^277^ Similarly, Singla’s team showed that ESC-derived exosomes could improve cardiac remodeling by enhancing anti-inflammatory M2 macrophages and reducing inflammation-induced pyroptosis.^278^ In terms of management of heart failure, Pang et al. exhibited that ESC-derived exosomes could attenuate heart failure, improve cardiac function and promote myocardial angiogenesis through the FGF2 signaling in a transverse aortic constriction-induced heart failure model.^279^ Using a coronary artery occlusion-induced heart failure model, Kervadec et al. showed that exosomes secreted by ESC-derived cardiovascular progenitors could recover cardiac functions such as reduced left ventricular end-systolic and end-diastolic volumes.^280^ Finally, the same research group later demonstrated that exosomes derived from more readily available cell sources, e.g., iPSCs, were capable of cardioprotective effects similar to those offered by ESC-derived ones.^281^

Other important subtypes of SC-Exo, such as iPSC-exo, HSC-exo and EPC-exo, have also exhibited cardio-protective effects. For example, exosomes secreted by iPSCs could exert cytoprotective effects on maintaining intracellular Ca^2+^ homeostasis and promoting cardiomyocyte survival, thereby improving recovery from MI.^282^ HSC-exo could reduce the cardiac injury-related indices and the degree of cardiac fibrosis while elevating the ejection fraction in an animal model of heart failure.^283^ In addition, systemic infusion of HSC-derived exosomes could improve ischemic cardiomyopathy in a rat model of acute MI, with additional benefits in treating the side effects such as kidney damage.^284^ Modification of HSCs using sonic hedgehog (Shh), an angiogenic factor, could preserve cardiac function after acute MI by delivery of exosomal Shh to ischemic myocardium.^285^ Ke et al. proved that EPC-derived exosomes could enhance the proliferation and angiogenesis of cardiac fibroblasts by activating mesenchymal-endothelial transition and decreasing the expression of HMGB1^286^ and later revealed that the exosomal miR-218-5p and miR-363-3p from EPC-derived exosomes could ameliorate MI by targeting the p53/JMY pathway.^287^ In an interesting study by Yue et al., IL-10 deficiency-induced systemic inflammation was found to compromise the reparative properties of EPC-derived exosomes on myocardial repair by upregulating integrin-linked kinase (ILK) enrichment in exosomes, and ILK-mediated activation of NF-κB pathway in recipient cells.^288^

In terms of treatment of thoracic disorders, Liu et al. proved that human ESCs-derived exosomes could alleviate inflammation, prevent excessive collagen deposition and preserve alveolar architecture in the lungs of mice with bleomycin-induced pulmonary fibrosis.^289^ This was achieved by the exosomal mi-17-5p targeting thrombospondin-2. Similarly, Zhou et al. demonstrated that the exosomal miR-302a-3p from iPSC-derived exosomes could suppress M2 macrophages via TET1, thereby mitigating pulmonary fibrosis.^290^ Liu et al. showed that EPC-derived exosomes could inhibit pulmonary artery smooth muscle cells proliferation and their resistance to apoptosis by regulating the Mitofusin-2 and Ras-Raf-ERK1/2 pathways, thereby acting as a potential therapeutic candidate for the treatment of pulmonary arterial hypertension.^291^ Two independent teams both revealed that human EPC-derived exosomes could improve outcomes of the LPS-induced acute lung injury partially through the delivery of miR-126 into the injured alveolus.^292,293^ Zhang et al. found that EPC-derived exosomes could improve the bioactivity of pulmonary microvascular endothelial cells and protect them from hyperoxic injury in the developing lung vasculature, thereby contributing to the treatment of bronchopulmonary dysplasia.^294^ Moreover, Montay-Gruel et al. demonstrated that human ESC-derived exosomes could improve the adverse late normal tissue complications associated with exposure of the lungs to ionizing radiation, such as those encountered during postoperative treatment of lung cancer.^295^

## Urology and SC-Exo therapy

Chronic kidney disease (CKD) is a syndrome characterized by persistent changes in kidney structure, function, or both, affecting 10−14% of the global population.^296^ The most common pathological feature and final manifestation of CKD is some form of renal fibrosis. Kidney fibrosis occurs when wound healing is deregulated, which leads to excessive accumulation of ECM proteins, such as collagen and fibronectin. In their study, Liu et al. discovered that bone marrow MSC-derived exosomes could alleviate vascular calcification, a detrimental indicator of morbidity and mortality in CKD.^297^ This was achieved through exosomal miR-381-3p by targeting NFAT5, which was further verified in severe arterial calcification in dialysis patients. In alternative studies, several groups demonstrated the capacity of bone marrow MSC-derived exosomes in treating renal fibrosis, each with a distinct mechanistic interpretation. In a cellular study, Yin et al. found that exosomes could prevent TGF-β1-induced epithelial-mesenchymal transition of renal tubular epithelial cells by transporting Nedd4L, which activates autophagy. In a 5/6 subtotal nephrotomy rat model, Liu et al. revealed that exosomes could improve renal function and reduce fibrotic size by regulating the Smurf2/Smad7 axis.^298^ In a unilateral ureteral occlusion-induced interstitial fibrosis mouse model, Lu et al. demonstrated that exosomes could improve renal fibrosis by reducing the polarization of M1 and M2 macrophages by activating EP2 receptors.^299^

Acute kidney injury (AKI) and CKD are closely connected, with each a risk factor for developing the other. Renal IRI is a leading cause of AKI and acute kidney failure.^300^ Lim et al. proved that exosomes from iPSC-derived MSCs could correct serum creatinine level, tubular necrosis, apoptosis, inflammatory cytokine production, and oxidative stress in AKI mice by activating the ERK1/2 signaling pathway.^301^ In a similar work by Zhang et al., the exosomal miR-21-5p from EPCs were found to alleviate sepsis-induced AKI by inhibiting RUNX1 expression in CLP rats.^302^

## Otorhinolaryngology and head & neck surgery and SC-Exo therapy

Hearing loss is the most common sensory deficit worldwide, affecting nearly 20% of the global population.^303^ The causes of sensorineural hearing loss (SNHL) can be very diverse, such as presbycusis, ototoxic medication-induced, noise-induced, and idiopathic sudden SNHL. Tsai’s team demonstrated that umbilical cord MSC-derived exosomes could rescue the loss of outer hair cells and repair cochlear damage in cisplatin-induced hearing loss.^304^ The underlying mechanism for the cochlea-protective effects is mediated by the miRNAs (e.g., miR-125a-5p and miR-125b-5p) and remodeling factors (e.g., fibronectin and galectin-3).

Cochlear IRI is one of the main reasons for idiopathic sudden and noise-induced SNHL, which can lead to irreversible damage of sensory hair cells and bipolar cochlear spiral ganglion.^305^ Its pathophysiology includes oxidative stress, excess cell death and dysregulated inflammation. Hao et al. discovered that exosomes derived from miR-21-overexpressing NSCs could prevent hearing loss from IRI by inhibiting the inflammatory process in the mouse cochlea.^306^ This was evidenced by a reduced auditory brainstem response threshold, upregulated IL-10 and downregulated TNF-α and IL-1β.

Hypothyroidism is a very common disease which could result from thyroidectomy or radioactive ablation to treat hyperthyroidism or thyroid cancer.^307^ Stem cell therapy and stem cell-derived exosome therapy have emerged as a promising management for hypothyroidism through thyroid regeneration. Using an in vitro culture system of thyroid lobes, Degosserie et al. suggested that EPC-derived exosomes could facilitate thyrocyte organization into thyroid follicles and lumen expansion (i.e., folliculogenesis), which was promoted by laminin-α1.^308^

Temporomandibular joint (TMJ) disorders are the second most common musculoskeletal condition affecting 31% of adults and 11% of children.^309^ Like other synovial joints in the body, TMJ is also prone to OA, sharing common pathophysiological processes (Section 3.2). Zhang and co-workers proved that MSC-derived exosomes could alleviate TMJ OA in an immunocompetent rabbit model by attenuating inflammation and restoring matrix homeostasis.^310^ The exosome-mediated joint repair was attributed to adenosine activation of Akt, ERK and AMPK signaling, as well as enhanced s-GAG synthesis.

## Ophthalmology and SC-Exo therapy

Acquired optic neuropathy is a major cause of blindness in adults, and has various etiologies, such as vascular, inflammatory, traumatic, toxic, compressive, and nutritional etiologies. Retinal ganglion cell (RGC) loss is the hallmark of optic neuropathies, via multiple cell death pathways.^311^ Mead and Tomarev found that bone marrow MSC-derived exosomes could promote survival of RGCs, and regeneration of their axons, in a rat optic nerve crush model.^312^ The exosomal neuroprotective and neuritogenic effects were accomplished through exosomal miRNAs, demonstrating a cell-free potential for traumatic and degenerative ocular diseases.

Retinal degenerative diseases (e.g., age-related macular degeneration and retinitis pigmentosa) are the leading cause of bilateral irreversible vision loss worldwide.^313^ It is characterized by progressive degeneration of photoreceptors, RGCs or retinal pigment epithelium (RPE) cells. Bian’s team discovered that exosomes derived from NSCs could preserve photoreceptors, visual function and prevent thinning of the outer nuclear layer in an RCS retinal degeneration rat model.^314^ This was achieved by marked inactivation of microglial inflammation via exosomal targeting of TNF-α, IL-1β and COX-2. In two consecutive studies, Gao’s team showed that ESC-derived exosomes could alleviate retinal degeneration by enhancing the proliferation and retrodifferentiation of retinal Müller cells as replacement retinal neuronal precursors. On one hand, this was accomplished by regulating the expression of Oct4 in Müller cells through exosomal HSP90.^315^ On the other hand, activation of the Wnt signaling pathway by delivering BDNF protein to Müller cells also played an important role.^316^ Furthermore, using a rat model of inherited retinal degeneration, Park et al. demonstrated that both subretinal and intravitreal injection of human HSC-derived exosomes could provide functional rescue of a degenerating retina.^317^

Failed healing of corneal defect often leads to corneal blindness which has been reported as second only to cataract in the leading causes of blindness.^318^ The most severe and recalcitrant cases would need corneal transplantation. Wang et al. compared exosomes derived from iPSCs and MSCs as therapeutic providers for the treatment of corneal epithelial defects.^319^ It was found that both types of exosomes could promote proliferation, cell cycle progression and migration while inhibiting apoptosis in vitro, and accelerate corneal epithelium defect healing in vivo. More importantly, the iPSC-derived exosomes had a stronger therapeutic effect than the MSC-derived exosomes.

Corneal transplantation is one of the most successful forms of solid organ transplantation. However, graft rejection can occur in up to 90% of high-risk recipients.^320^ Both innate and adaptive immunity are the predominant reason for graft failure. Immunosuppressive drugs have shown only partial effectiveness. Jia et al. showed that MSC-derived exosomes could cross biological barriers and prolong graft survival time in a rat model of corneal allograft rejection.^321^ This was likely caused by inhibition of the infiltration of CD4^+^ and CD25^+^ T cells and the reduction of IFN-γ and CXCL11 via the Th1 signaling pathway.

## Obstetrics and gynecology and SC-Exo therapy

Primary ovarian insufficiency (POI), or premature ovarian failure (POF), is defined as loss of ovarian function before the age of 40. Non-genetic causes of POI include autoimmune disorders, metabolic conditions, infections, and iatrogenic procedures (e.g., chemotherapy, radiotherapy, and surgery). Women with POI suffer from various complications, such as osteoporosis, infertility, cardiovascular disorders and depression.^322^ Although promptly initiating hormone replacement therapy is critical to control these symptoms and complications, it fails to restore ovarian function. Currently-tested experimental therapies include mitochondrial activation, in vitro activation, stem cell therapy, and exosome therapy.^323^

The work conducted by Li et al. showed that umbilical cord MSC-derived exosomes could improve ovarian function in a cyclophosphamide (CTX)-induced POI mouse model.^324^ The SC-exo therapy not only restored ovarian function-related hormone levels and the number of ovarian follicles, but also improved the reproductive ability of POI mice. In addition, the exosomes promoted the proliferation of ovarian granulosa cells (GCs) by regulating the Hippo pathway, and the effect was neutralized by a YAP inhibitor. Similar results were obtained using exosomes from iPSC-derived MSCs.^325^ The work performed by Ding et al. illustrated that umbilical cord MSC-derived exosomes could restore ovarian phenotype and function in a POI mouse model, promote proliferation of CTX-damaged human GCs and oocytes, and alleviate ROS accumulation by delivering exosomal miR-17-5p and targeting its downstream mRNA SIRT7.^326^ It was further elucidated that miR-17-5p down-regulated PARP1, γH2AX, and XRCC6 expression by inhibiting SIRT7. Lastly, the work completed by Yang et al. revealed that bone marrow MSC-derived exosomes could recover the estrus cycle, increase the number of basal and sinus follicles, increase estradiol E2 and anti-Mullerian hormone levels, and reduce follicle stimulating hormone and luteinizing hormone levels in a chemotherapy-induced POF rat model.^327^ Mechanistically, this was achieved by exosomal miR-114-5p that targets PTEN.

## From preclinical studies to clinical trials of exosome therapy

Many preclinical studies, as discussed in Sections 3 to 11, have confirmed the advantages of MSC-derived and NSC-derived exosomes to treat many diseases spanning the subspecialties of surgical practice. Without restricting the scope to stem cell-derived exosomes only, many clinical trials have demonstrated the role of exosomes to be twofold: biological markers and therapeutic agents. A search on ClinicalTrials.gov using ‘exosome therapy’, ‘exosome treatment’, and ‘exosome’ as keywords generated 188 records. However, only 60 (32%) of these directly relate to interventional studies using exosomes as therapeutic agents (Table 5). The rest, especially oncology-related trials, mostly used exosomes as biomarkers, such as key players during disease pathogenesis (e.g., NCT04288141, NCT04154332), diagnostic markers and guidance before treatment (e.g., NCT04629079, NCT03791073, NCT05451342, NCT03432806), monitoring indices and predictive tools for treatment efficiency (e.g., NCT05427227, NCT04499794, NCT04852653, NCT05370105, NCT03800121, NCT05370105, NCT05328089), and prognostic indicators after treatment (e.g., NCT06026735, NCT05705583, NCT05411445, NCT04167722, NCT05575622). The clinical applications of exosomes as biomarkers have been extensively explored in other reviews^328–332^ and are therefore beyond discussion in this review. In terms of the clinical characteristics of the 60 clinical trials on exosome therapy (Table 5), there are several highlights worth mentioning.

**Table 5:** Clinical trials of exosome therapy

Category of conditions	Specific disease	NCT number	Source of exosome	Gender	Age	Phases	Enrollment	Country
Behavior & mental disorders	depression & anxiety	NCT04202770	MSC	all	>18	N/A	300	USA
Blood & lymph conditions	coagulopathy	NCT02594345	red blood cell	all	18–80	N/A	18	Germany
Digestive system diseases	perianal fistula	NCT05499156	MSC	all	18–70	1/2	80	Iran
		NCT05402748	MSC	all	18–70	1/2	80	Iran
	liver cirrhosis	NCT05871463	MSC	all	18–75	2	15	Iran
	IBS	NCT04879810	MSC	all	>18	N/A	4	USA
Diseases at or before birth	ELBW birth	NCT05490173	MSC	all	1–3 days	N/A	10	Russia
Eye diseases	dry eye disease	NCT05738629	MSC	all	18–70	1/2	12	China
		NCT04213248	MSC	all	18–70	1/2	27	China
	macular holes	NCT03437759	MSC	all	<80	early 1	44	China
	retinitis pigmentosa	NCT05413148	MSC	all	18–70	2/3	135	Turkey
Gland & hormone-related diseases	T1DM	NCT02138331	MSC	all	18–60	2/3	20	Egypt
Heart & blood diseases	MI	NCT05669144	MSC, mitochondria	all	35–80	1/2	20	Iran
	aortic dissection	NCT04356300	MSC	all	20–80	N/A	60	China
Mouth & tooth diseases	periodontitis	NCT04270006	MSC	all	18–50	early 1	10	Egypt
	oral mucositis	NCT01668849	plant, grape	all	20–85	1	60	USA
Musculoskeletal diseases	meniscal injury	NCT05261360	MSC	all	30–50	2	30	Turkey
	OA, knee	NCT05060107	MSC	all	30–70	1	10	Chile
	degenerative disc disease	NCT04849429	PRP	all	18–60	1	30	India
	bone loss	NCT04998058	MSC	all	>35	1/2	20	Brazil
Neoplasms	metastatic pancreas cancer	NCT03608631	MSC + KRAS G12D siRNA	all	>18	1	28	USA
	colon cancer	NCT01294072	plant, curcumin	all	>20	1	35	USA
	NSCLC	NCT01159288	tumor antigen-loaded DC	all	18–70	2	41	France
	bladder cancer	NCT05559177	chimeric exosomal tumor vaccines	all	18–85	early 1	9	China
	HCC	NCT05375604	CDK-004	all	>18	1	9	USA
Nervous system diseases	focal epilepsy	NCT05886205	iPSC	all	18–70	early 1	34	China
	craniofacial neuralgia	NCT04202783	N/A	all	>18	N/A	100	USA
	ischemic stroke	NCT03384433	MSC	all	40–80	1/2	5	Iran
	AD	NCT04388982	MSC	all	>50	1/2	9	China
Nutritional & metabolic diseases	familial hypercholesterolemia	NCT05043181	LDLR mRNA delivery	all	18–45	1	30	China
Respiratory tract diseases	COVID-19	NCT04276987	MSC	all	18–75	1	24	China
		NCT05787288	MSC	all	18–75	early 1	240	China
		NCT05808400	MSC	all	18–80	early 1	80	China
		NCT05216562	MSC	all	18–75	2/3	60	Indonesia
		NCT04602442	MSC	all	18–65	2	90	Russia
		NCT04491240	MSC	all	18–65	1/2	30	Russia
		NCT04493242	MSC	all	18–85	2	102	USA
		NCT04798716	MSC	all	>18	1/2	55	USA
		NCT05387278	MSC	all	18–75	1	20	USA
		NCT04389385	COVID-19 specific T cell	all	18–75	1	60	Turkey
		NCT04747574	EXO-CD24	all	18–85	1	35	Israel
		NCT04969172	EXO-CD24	all	18–80	2	155	Israel
		NCT04902183	CovenD24	all	18–80	2	90	Greece
		NCT04384445	Zofin	all	>18	1/2	20	USA
		NCT04657406	Zofin	all	>18	N/A	N/A	USA
		NCT05228899	Zofin	all	>18	1/2	30	USA
	ARDS	NCT04602104	MSC	all	18–70	1/2	169	China
		NCT05354141	MSC	all	18–65	3	970	USA
		NCT05947747	EXO-CD24	all	>18	2	90	Israel
	COPD	NCT05643729	Zofin	all	40–80	1/2	20	USA
	pulmonary infection, drug-resistant	NCT04544215	MPC	all	18–75	1/2	60	China
Skin & connective tissue diseases	atopic dermatitis	NCT05969717	iPSC	all	18–70	early 1	20	China
	psoriasis	NCT05523011	MSC	all	>21	1	10	Singapore
	dystrophic epidermolysis bullosa	NCT04173650	MSC	all	>6	1/2	10	USA
	skin aging	NCT05813379	MSC	female	35–65	1/2	20	Iran
	chronic ulcer	NCT04134676	MSC-CM	all	18–80	1	38	Indonesia
	cutaneous wound	NCT02565264	plasma	all	all	early 1	5	Japan
		NCT05475418	MSC	all	18–60	N/A	5	China
	alopecia	NCT05658094	MSC	all	25–65	N/A	20	Iran
Urinary tract & sexual organs conditions	PCOS	NCT03493984	plant, ginger & aloe	female	18–40	N/A	N/A	USA

Data obtained from ClinicalTrials.gov using ‘exosome therapy’, ‘exosome treatment’, and ‘exosome’ as keywords, as of 2023-09-08. The categorization of diseases follows the system by Clinical Trials.gov

*AD* Alzheimer’s disease, *ARDS* acute respiratory distress syndrome, *CM* conditioned medium, *COPD* chronic obstructive pulmonary disease, *DC* dendritic cell, *DM* diabetes mellitus, *ELBW* extremely low birth weight, *HCC* hepatocellular carcinoma, *IBS* irritable bowel syndrome, *iPSC* induced pluripotent stem cell, *LDLR* low-density lipoprotein receptor, *MI* myocardial infarction, *MPC* mesenchymal progenitor cell, *MSC* mesenchymal stem cell, *NSCLC* non-small cell lung cancer, *OA* osteoarthritis, *PCOS* polycystic ovary syndrome, *PRP* platelet-rich plasma, *siRNA* small interfering RNA

Firstly, the spectrum of diseases covered is very broad, as both surgical and medical conditions are included. These include many surgical disorders discussed in Sections 3 to 11, such as orthopedic diseases (osteoarthritis of the knee in NCT05060107, bone loss in NCT04998058, and intervertebral disc degeneration in NCT04849429), neurosurgical diseases (ischemic stroke in NCT03384433 and Alzheimer’s disease in NCT04388982), plastic surgical diseases (cutaneous wound healing in NCT02565264 and NCT05475418), general surgical diseases (liver cirrhosis in NCT05871463), cardiothoracic diseases (myocardial infarction in NCT05669144), and ophthalmology diseases (retinitis pigmentosa in NCT05413148). In other words, some preclinical studies have not yet developed into clinical trials. These include, but are not limited to, exosome therapy for fracture^333^ spinal cord injury^334^ traumatic brain injury^335^ acute liver injury^336^ and hearing loss^304,306^ which might serve as future directions for exosome therapy-related clinical trials.

Secondly, an increasing number of clinical trials focused on two medical conditions, i.e., COVID-19 (16 trials, 27%) and cancer (5 trials, 8%). MSC-derived exosomes can manage viral infection and lung damage in COVID-19 through both reparative actions and regenerative effects.^337^ The former manifests as blockage of viral entry and replication, and suppression of the cytokine storm, whereas the latter as prevention of inflammation, and fluid-clearance and restoration of lung permeability. In addition, exosomes can be engineered into a drug (e.g., CD24, a potent immune regulator) delivery platform^338^ and even a vaccine^339^ to combat COVID-19. In contrast to the MSC-derived exosomes for COVID-19 management, exosomes used for cancer treatment mainly rely on non-stem cells and cargo engineering. This is partially because MSC-derived exosomes demonstrate controversial effects on tumorigenesis and metastasis.^340,341^ Although the exact interaction between MSC-derived exosomes and tumor cells remains open to debate, scientists have overcome several obstacles by modifying exosomal cargos to deliver antioncogenic nucleic acids and anticancer medications, and exosomal membranes for specific tumor targeting.^342–346^

Finally, among the 40 clinical trials using stem cell-derived exosomes for disease treatment, 38 (95%) used MSC and 2 (5%) used iPSC as the cellular source for exosomes. However, this differs significantly from the preclinical studies discussed in Sections 3 to 11. The lack of use of NSC-derived exosomes in clinical trials might be partially because of the supply constraints of their parental cells.^347^ Currently, NSCs can be obtained from three sources^348^ (Fig. 1a): 1, isolation from primary CNS tissues (e.g., adult and fetal brain); 2, differentiation from pluripotent stem cells (e.g., iPSCs and ESCs); 3, reprogramming of somatic cells (e.g., fibroblasts and blood cells) to iNSCs.^349^ Recent studies have developed fibroblast-derived iNSCs, opening a new window for obtaining exosomes from NSC-like cells. These iNSC-exo could not only promote cell survival and proliferation no less than NSC-exo in vitro^350,351^ but also enhance recovery after ischemic stroke^148^ and mitigate AD-like phenotypes in preclinical models.^190^ Therefore, iNSCs might be an excellent cellular source to produce clinical-grade exosomes in clinical trials.

In summary, the progression from preclinical studies to clinical trials of exosome therapy has been expeditious. However, issues like insufficient clinical indications for exosome treatment and limited sources for parental stem cells remain to be addressed. In addition, once the limitations (Table 1) in upscaling of manufacturing, compliance with good manufacturing practice, and regulatory framework are overcome^329^ stem cell-derived exosome therapy will soon be incorporated into clinical practice and serve at the patient’s bedside.

## Conclusion and future perspectives

Exosomes have been pursued recently as a cell-free alternative to stem cell-based therapy. ESC-, iPSC-, HSC-, MSC-, NSC- and EPC-derived exosomes are of particular interest, partially due to the pluripotency or multipotency of their parental cells. After going through production and purification with or without modification, stem cell-derived exosomes have demonstrated tremendous potential in treating numerous diseases encountered during surgical practice. These are exemplified by disorders in orthopedic surgery (e.g., fracture, osteoarthritis, and spinal cord injury); neurosurgery (e.g., ischemic stroke, traumatic brain injury, and Alzheimer’s disease); plastic surgery (e.g., wound healing); general surgery (e.g., acute liver injury); cardiothoracic surgery (e.g., myocardial infarction); urology (e.g., chronic kidney disease); head and neck surgery (e.g., sensorineural hearing loss); ophthalmology (e.g., acquired optic neuropathies), and gynecology (e.g., primary ovarian insufficiency). Mechanistically, the diverse therapeutic effects of stem cell-derived exosomes are achieved through disease-specific cellular and tissue responses (e.g., tissue regeneration, anti-inflammation, anti-cell death, immunomodulation, and anti-oxidative stress) and tissue-specific molecular signaling pathways (e.g., Wnt/β-catenin, PTEN/PI3K/Akt/HIF-1α, MAPK, and JAK/STAT pathways). Collectively, stem cell-derived exosome therapy has been proven to be a potent and versatile surrogate to stem cell therapy in the surgical arena.

Future emphasis of clinical applications of stem cell-derived exosomes should be placed on various nodes of this therapeutic pipeline. Firstly, targeting the pretherapeutic large-scale production of exosomes, a high-throughput cellular source as well as a reproducible and scalable production and isolation protocol are required. Compared to the static system growing monolayer cells, dynamic system in the form of bioreactor, e.g., hollow-fiber bioreactor and stirred tank bioreactor, can increase the efficiency by producing copious cells and exosomes in a short period of time. However, the phenotype of parental cells and derived exosomes might change due to physical and shear stress encountered in a reactor. Thus, the working parameters of the bioreactor must be optimized to facilitate large-scale production of stem cell-derived exosomes. Secondly, targeting the therapeutic modality of exosomes, delivery methods other than systemic administration need to be explored. When delivered through the venous system, exosomes are rapidly cleared from blood circulation and accumulate in the liver, spleen and lungs, which can be overcome by local delivery. Various biomaterials have been recently used to protect, assist and augment locally delivered exosomes to maximize their therapeutic effects. These biomaterials could be designed according to their sources (e.g., natural, synthetic, and hybrid polymers), format (e.g., scaffold, patch, spray, and microneedle), and responsiveness (e.g., temperature, pH, and protein), thereby allowing disease-specific customization. Lastly, targeting the therapeutic indications of exosome therapy, more diseases than the ones discussed in this review should be included into future preclinical studies and clinical trials. For example, airway inflammatory conditions (e.g., allergic rhinitis and asthma) could be suitable candidates for exosome therapy, considering the immunomodulatory effect of MSC-derived exosomes. Disorders that are best treated using surgical implants (e.g., cochlear implant, intraocular lenses, and contraceptive intrauterine devices) could be managed in the form of implant-based local release of exosomes. In addition to primary diseases, secondary conditions including surgical operation- and general anesthesia-related complications (e.g., cognitive impairment, wound paresthesia, and malignant hyperthermia) might become therapeutic targets of exosome therapy. Collectively, efforts to upscale exosome production in conjunction with multimodal exosome delivery will accelerate the clinical applications of stem cell-derived exosomes in a rapidly expanding disease spectrum.
